# Association of dietary patterns with chronic respiratory health among U.S. adults

**DOI:** 10.3389/fimmu.2024.1457860

**Published:** 2024-12-06

**Authors:** Hui Li, XiaoLi Tang, XinWei Guo, MingZhe Zhang, MingJie Zhang, JiaQi Nie, SanYou Fang, Hong Zhang, Yuanmei Shi, Xiaorong Dai, JiaQi Li, Xin Yin

**Affiliations:** ^1^ Department of Medical, Taixing People's Hospital, Taixing, China; ^2^ School of Public Health, Wuhan University, Wuhan, China; ^3^ Department of Health Promotion, XiaoGan Center For Disease Control and Prevention, Xiaogan, China; ^4^ Department of Medical Optics, Hospital of Stomatology Wuhan University, Wuhan, China

**Keywords:** HEI-2020, DII, Mediterranean diet, DASH diet, chronic respiratory diseases, NHANES

## Abstract

**Background:**

Respiratory health is closely related to immune system function, and diet can also influence immune homeostasis. Diet, an important part of a healthy lifestyle, is also linked to respiratory health. We aimed to explore the relationship between different dietary patterns and the risk of chronic respiratory diseases (CRDs), including chronic bronchitis (CB), emphysema, and asthma.

**Method:**

A total of 23,042 adults from the United States were selected from the National Health and Nutrition Examination Survey (NHANES) dataset between 2007 and 2018. Diet quality was assessed using 2-day, 24-hour dietary recall data and quantified as the Healthy Eating Index-2020 (HEI-2020), the Dietary Inflammation Index (DII), the Mediterranean Dietary Index (MEDI), and the Dietary Approaches to Stop Hypertension Index (DASHI). Binary logistic regression models, restricted cubic splines (RCS), and the weighted quartile sum (WQS) models were used to assess the relationship between diet quality and the risk of CB, emphysema, and asthma.

**Results:**

In logistic regression analyses of the four dietary indices with the three chronic respiratory diseases, it was consistently observed that higher dietary quality scores were linked to a reduced risk of respiratory disease. These consistent trends were also evident in the assessments of the dose–response relationship between dietary quality score and the risk of respiratory disease. Furthermore, evaluations of the combined effects of dietary components across different dietary indices in the risk of chronic respiratory disease yielded results consistent with the logistic regression models. Notably, high-quality protein, minerals, and fiber-rich fruits and vegetables emerged as the food groups making the most significant contributions to health across different dietary indices.

**Conclusion:**

Low-quality diets, lacking in high-quality protein, minerals, and fruits and vegetables rich in dietary fiber, are associated with a higher risk of chronic respiratory disease, regardless of the dietary index used to measure diet quality.

## 1 Introduction

Chronic respiratory diseases are significant public health issues in the United States (U.S.) and even globally, with chronic bronchitis (CB) (1), emphysema (2), and asthma (3) being the most common (4, 5). Approximately 8% of U.S. adults have asthma (6). CB and emphysema are both manifestations of chronic obstructive pulmonary disease (COPD), which affects more than 15 million people in the U.S. and is the fourth leading cause of death in the U.S. and the third leading cause of death globally (6–9).

The human respiratory tract is constantly exposed to harmful microorganisms and air pollutants, and the immune system responds to these pests to protect the host. However, the production of an unbalanced inflammatory response by the immune system may itself promote tissue damage and ultimately lead to acute and chronic respiratory diseases (10–12). When damage to the respiratory tract occurs, the respiratory epithelium produces a series of mediators that protect respiratory health by directly killing microorganisms, activating tissue-resident immune cells, and recruiting leukocytes from the bloodstream. The mediators are composed of a large number of leukocytes, including innate lymphocytes (ILCs), which are actively involved in the pathogenesis of chronic respiratory diseases (13, 14). Regarding the prevention and management of chronic respiratory diseases (12, 15), notable treatment guidelines emphasize the significance of adopting a healthy lifestyle as a pivotal measure to prevent diseases and enhance patients’ quality of life. Consequently, beyond the use of relevant therapeutic medications, prioritizing a healthy lifestyle is of paramount importance (16, 17).

Diet is an important part of a healthy lifestyle, and in recent decades, there has been an increasing number of studies on diet and chronic diseases (18–21), such as cognitive, metabolic, and cardiovascular diseases. Diet is also closely related to immunity. Dietary fat provides calories and ATP in the non-specific immunity of leukocytes (22). Diet and nutritional status are also major regulators of memory T-cell biology and organismal health (23). Studies have shown that healthy diets contribute to the stability of immune system function, while unhealthy diets such as high-salt diets and high-calorie diets can induce immune dysfunction (24, 25). The Healthy Eating Index (HEI) serves as a valuable indicator of dietary quality, aligning with the Dietary Guidelines for Americans (DGA) (26). In addition to the HEI, other indices contribute to our understanding of diet’s effects on health. The Dietary Inflammation Index (DII) gauges the influence of diet on the body’s inflammatory response (27). Meanwhile, the Mediterranean Dietary Index (MEDI) and the Dietary Approaches to Stop Hypertension Index (DASHI) have been used to represent the Mediterranean dietary pattern and the Dietary Approaches to Stop Hypertension (DASH) dietary pattern to assess the quality of healthy diets (28, 29). These dietary indices (DII, MEDI, and DASHI) better reflect the dietary intake of the population and are highly correlated with the human health levels and the risk of chronic diseases. However, there are no studies related to different dietary patterns and chronic respiratory diseases in U.S. populations. A healthy diet contributes to the homeostasis of the immune system. At the same time, the immune system is closely linked to respiratory health. However, few studies have examined the relationship between diet and respiratory and lung health. We hypothesized that improvements in dietary quality would contribute to the reduction of the risk of chronic respiratory diseases.

The National Health and Nutrition Examination Survey (NHANES) database is a large continuous cross-sectional survey in the U.S. We used this large, representative database of U.S. residents to explore the relationship between different dietary pattern indices and three chronic respiratory diseases. Because of the importance of chronic respiratory health and the indispensable role of diet in daily life, the study of dietary and respiratory health correlations explored in this study has strong public health implications.

## 2 Materials and methods

### 2.1 Study sample

**Figure 1:**
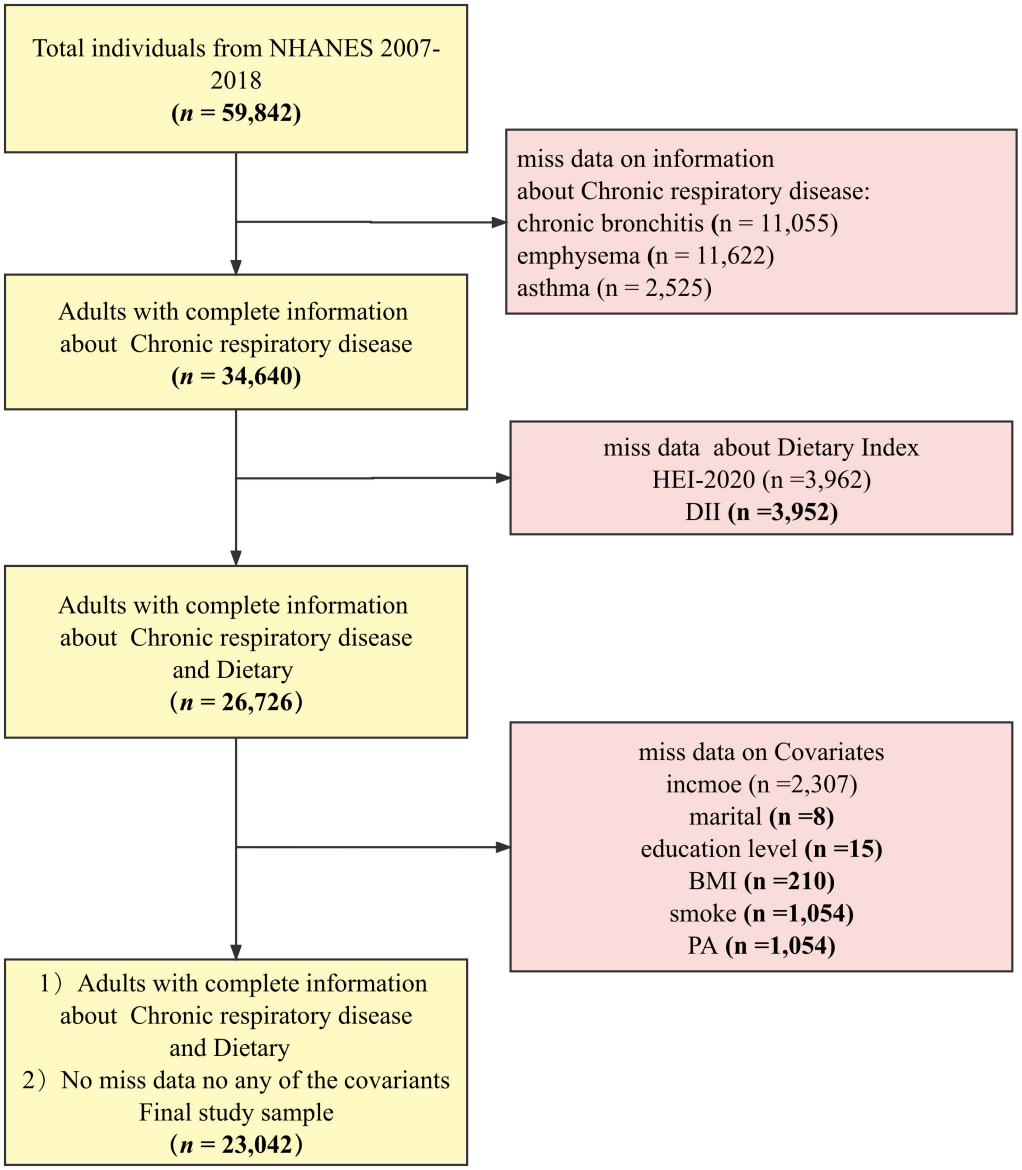
Flowchart.

The NHANES database is a regularly conducted cross-sectional study from the Centers for Disease Control and Prevention’s (CDC’s) National Center for Health Statistics (NCHS) that investigates nutritional intake and health-related conditions of populations in the U.S (30). The NHANES utilizes a complex, multistage sampling design to make the survey results well-representative of populations across the U.S. The data for this study were obtained from NHANES 2007–2018. Data with complete demographics (gender, age, education, and family income), behavior (BMI, physical activity, smoking, and drinking status), chronic disease data (diabetes and hypertension), and dietary data were included in the sample. The final sample (*n* = 23,042) was weighted to represent 160 million non-institutionalized adult U.S. population, and the process can be seen in **Figure 1**.

### 2.2 Diet quality

Our analysis is grounded in data from four widely recognized dietary pattern indices that serve as proxies for dietary quality: the HEI-2020, the DII, the MEDI, and the DASHI. The HEI-2020 evaluates adherence to the DGA 2020–2025, encompassing 13 components (31): adequacy components (total vegetables, greens and beans, total fruits, whole fruits, whole grains, dairy, total protein foods, seafood, plant proteins, and fatty acids) and moderation components (sodium, refined grains, saturated fats, and added sugars). Each component is weighted differently and has a distinct maximum score. HEI-2020 scores range from 0 to 100, with higher scores indicating superior diet quality. The DII was crafted to gauge the inflammatory impact of diets on the body. Given the constraints of the NHANES database, our study incorporated 28 dietary components for DII calculations, including vitamin A, vitamin C, vitamin D, B vitamins, proteins, dietary fiber, unsaturated fatty acids, and others. The DII is derived by calculating the *z*-value, which is the difference between the world average intake and the individual’s actual intake, divided by the standard deviation. To mitigate bias, this value is converted into percentile scores, doubled, and then adjusted by subtracting 1.0 to center the distribution to approximately 0. These centered scores are subsequently multiplied by the Inflammatory Effects Score, and the food parameters are summed for a comprehensive assessment. For detailed methodology, refer to the original research (32). The MEDI score, adapted from the Mediterranean diet scale by Trichopoulou et al. (33), comprises eight dietary components, namely, vegetables, fruits, nuts, whole grains, legumes, fish, the monounsaturated-to-saturated fat ratio (MtSR), and red and processed meats. A higher MEDI score signifies better diet quality (34). The DASHI measures the adherence to the DASH dietary patterns, and we have utilized the version validated by Mellen et al (35), which includes nine dietary components (total fat, saturated fat, protein, fiber, cholesterol, calcium, magnesium, sodium, and potassium). Higher DASHI scores are indicative of better diet quality. **Supplementary Table 1** provides further details on these dietary indices. For weighted Scott-Rao chi-square tests and weighted logistic regressions, the dietary index was treated as both a continuous and a categorical variable. The dietary index scores were categorized into quartiles, designated as Q1 (reference group), Q2, Q3, and Q4 (36).

### 2.3 Chronic respiratory diseases

Three chronic respiratory diseases (CB, emphysema, and asthma) were selected as outcome variables in this study.

#### 2.3.1 CB

All subjects were asked “Has a doctor or other health professional ever told you that you had chronic bronchitis?” and then categorized into non-patients (reference group) and patients according to the answers (37).

#### 2.3.2 Emphysema

All subjects were asked “Has a doctor or other health professional ever told you that you had emphysema?” and then categorized into non-patients (reference group) and patients according to the answers (37).

#### 2.3.3 Asthma

All subjects were asked “Has a doctor or other health professional ever told you that you had asthma?” and then categorized into non-patients (reference group) and patients according to the answers (37).

### 2.4 Covariates

Based on the relevant studies, the relevant covariates were also selected to correct the model in this study.

1. Demographic variables include gender (male and female), age (<40, between ≥40 and <60, and ≥60 years), race (non-Hispanic white, non-Hispanic black, Mexican, and other), education level (less than high school, high school graduate/GED or equivalent, and above), marital status (married or living with partner, divorced or widowed, and never married), and income level (1.3 < pir, 3.5 ≤ pir < 3.5, and 3.5 ≤ pir) (38–40).
2. Lifestyle variables include BMI, smoking, drinking, and physical exercise (PA). We used serum cotinine levels to reflect smoking status with tertile definition, and alcohol use data from dietary surveys to reflect drinking status. Metabolic equivalent (MET) was calculated to reflect physical activity (39, 41).
3. Disease covariates include hypertension and diabetes. Hypertension is defined using self-reporting in conjunction with blood pressure levels. Diabetes was defined using self-reporting in conjunction with plasma fasting glucose, glycohemoglobin, and oral glucose tolerance test (OGTT) (42, 43).

### 2.5 Statistical analysis

Baseline characteristics across various groups were analyzed utilizing chi-square tests for categorical variables and *t*-tests for continuous variables. Binary logistic regression models were applied to assess the correlation between distinct dietary index scores and the likelihood of developing chronic respiratory diseases, encompassing CB, emphysema, and asthma. The dose–response relationship between the scores of different dietary indices and the prevalence of chronic respiratory diseases was scrutinized with the aid of RCS. The WQS regression model was employed to evaluate the synergistic effects of dietary components from the various indices on the risk of chronic respiratory diseases. Baseline characteristics across various groups were analyzed utilizing chi-square tests for categorical variables and *t*-tests for continuous variables. Binary logistic regression models were applied to assess the correlation between distinct dietary index scores and the likelihood of developing chronic respiratory diseases, encompassing CB, emphysema, and asthma. The dose–response relationship between the scores of different dietary indices and the prevalence of chronic respiratory diseases was scrutinized with the aid of RCS. The WQS regression model was employed to evaluate the synergistic effects of dietary components from the various indices on the risk of chronic respiratory diseases (44).

The study sample was weighted and analyzed using the recommended appropriate methodology for a study population sample with a complex sampling design. This study also used sensitivity analyses with unweighted samples and gender subgroups.

All statistical tests were two-sided, and significance was considered at *p* < 0.05. All statistical analyses were performed with R (version 4.1.2). RCS was implemented with the R package “rms” (version 6.3-0). WQS was implemented with the R package “gWQS” (version 3.0.4).

## 3 Results

### 3.1 Characteristics of the baseline

Baseline characteristics are shown in **Table 1**. After screening the sample, a total sample of *n* = 23,042 was included, of which 47.3% were male and 52.7% were female; the mean score of HEI-2020 was 54.01 ± 13.42, the mean score of DII was 1.39 ± 1.59, the mean score of MEDI was 5.70 ± 1.01, and the mean score of DASHI was 3.52 ± 1.15. As seen in the baseline characterization between the healthy population and the chronic respiratory disease population, adults with chronic respiratory disease were more likely to be older, have lower income, be obese, be less physically active, have chronic illnesses, and have lower dietary quality.

**Table 1:** The baseline population characteristics among U.S. adults by the three chronic respiratory diseases.

Characteristics (weighted%)	Total	Asthma		*p*	Emphysema		*p*	Chronic bronchitis		*p*
		Non-patients	Patients		Non-patients	Patients		Non-patients	Patients	
	23,042	19,647 (85.1%)	3,395 (14.9%)		22,553 (98.2%)	489 (1.8%)		21,681 (94.3%)	1,361 (5.7%)	
Sex				<0.001			0.009			<0.001
Male	10,993 (47.3%)	9,619 (48.7%)	1,374 (39.4%)		10,703 (47.2%)	290 (54.9%)		10,504 (48.2%)	489 (33.4%)	
Female	12,049 (52.7%)	10,028 (51.3%)	2,021 (60.6%)		11,850 (52.8%)	199 (45.1%)		11,177 (51.8%)	872 (66.6%)	
Age (%)				<0.001			<0.001			<0.001
<40	7,535 (34.7%)	6,264 (33.8%)	1,271 (40%)		7,513 (35.3%)	22 (4.5%)		7,264 (35.5%)	271 (21.3%)	
Between ≥40 and <60	7,728 (38.4%)	6,623 (38.9%)	1,105 (35.2%)		7,602 (38.6%)	126 (25.9%)		7,241 (38.3%)	487 (39.5%)	
≥60	7,779 (26.9%)	6,760 (27.3%)	1,019 (24.8%)		7,438 (26.1%)	341 (69.6%)		7,176 (26.1%)	603 (39.2%)	
Race (%)				<0.001			<0.001			<0.001
Non-Hispanic white	10,282 (69.8%)	8,664 (69.7%)	1,618 (70.3%)		9,933 (69.5%)	349 (82.7%)		9,483 (69.3%)	799 (76.9%)	
Non-Hispanic black	4,696 (10.2%)	3,898 (9.9%)	798 (12.1%)		4,640 (10.3%)	56 (5.1%)		4,442 (10.3%)	254 (9.4%)	
Mexican	3,289 (7.8%)	2,987 (8.2%)	302 (5%)		3,273 (7.9%)	16 (1.2%)		3,187 (8%)	102 (3.7%)	
Other	4,775 (12.3%)	4,098 (12.2%)	677 (12.6%)		4,707 (12.3%)	68 (11.1%)		4,569 (12.4%)	206 (9.9%)	
Education (%)				0.423			<0.001			<0.001
Less than high school	5,048 (14%)	4,363 (14.1%)	685 (13.4%)		4,853 (13.6%)	195 (31.3%)		4,706 (13.7%)	342 (18.3%)	
High school graduate/GED or equivalent	5,237 (22.6%)	4,479 (22.7%)	758 (21.8%)		5,105 (22.5%)	132 (29.3%)		4,881 (22.3%)	356 (27.4%)	
Above	12,757 (63.4%)	10,805 (63.2%)	1,952 (64.8%)		12,595 (63.9%)	162 (39.4%)		12,094 (64%)	663 (54.3%)	
Income (%)				<0.001			<0.001			<0.001
1.3 < pir	7,169 (20.5%)	5,900 (19.6%)	1,269 (25.9%)		6,934 (20.2%)	235 (37.2%)		6,585 (19.9%)	584 (31.2%)	
3.5 ≤ pir < 3.5	8,688 (35.4%)	7,525 (35.7%)	1,163 (33.4%)		8,502 (35.3%)	186 (41.4%)		8,171 (35.1%)	517 (39.4%)	
3.5 ≤ pir	7,185 (44.1%)	6,222 (44.7%)	963 (40.7%)		7,117 (44.5%)	68 (21.4%)		6,925 (45%)	260 (29.3%)	
Marital status (%)				<0.001			<0.001			<0.001
Married	13,946 (65.1%)	12,101 (65.9%)	1,845 (60.4%)		13,704 (65.1%)	242 (65.3%)		13,252 (65.5%)	694 (58.5%)	
Divorced or widowed	5,060 (17.9%)	4,225 (17.5%)	835 (20%)		4,843 (17.9%)	217 (17.4%)		4,588 (17.2%)	472 (28.5%)	
Never married	4,036 (17.1%)	3,321 (16.6%)	715 (19.7%)		4,006 (17.1%)	30 (17.3%)		3,841 (17.3%)	195 (13%)	
BMI [mean (SD)]	29.22 (6.90)	29.15 (6.78)	31.02 (8.21)	<0.001	29.21 (6.89)	29.55 (7.53)	0.519	29.08 (6.79)	31.44 (8.24)	<0.001
PA [mean (SD)]	592.43 (417.22)	545.33 (419.37)	569.76 (446.4)	0.208	594.37 (419.28)	488.24 (264.59)	<0.001	597.28 (420.29)	512.47 (353.53)	<0.001
Smoking status (%)				<0.001			<0.001			<0.001
T1	1,064 (6.7%)	920 (6.9%)	144 (6%)		1,052 (6.9%)	12 (2.3%)		1,014 (6.6%)	50 (4.6%)	
T2	7,651 (46.3%)	6,620 (47.3%)	1,031 (41.1%)		7,545 (46.8%)	106 (24.4%)		7,289 (47.6%)	362 (33.3%)	
T3	7,687 (46.9%)	6,323 (45.8%)	1,364 (52.9%)		7,382 (46.3%)	305 (73.2%)		7,011 (45.8%)	676 (62.1%)	
Drinking status [mean (SD)]	0.34 (0.47)	0.29 (0.46)	0.28 (0.45)	0.476	0.34 (0.47)	0.22 (0.42)	<0.001	0.34 (0.47)	0.27 (0.45)	<0.001
Diabetes (%)				0.003			<0.001			<0.001
No	19,258 (87.6%)	16,520 (87.9%)	2,738 (85.6%)		18,924 (87.9%)	334 (70.7%)		18,270 (88.3%)	988 (76.6%)	
Yes	3,784 (12.4%)	3,127 (12.1%)	657 (14.4%)		3,629 (12.1%)	155 (29.3%)		3,411 (11.7%)	373 (23.4%)	
Hypertension (%)				0.109			<0.001			<0.001
No	12,050 (0%)	10,362 (58.1%)	1,688 (56%)		11,914 (58.3%)	136 (30.2%)		11,553 (0%)	497 (0%)	
Yes	10,992 (42.2%)	9,285 (41.9%)	1,707 (44%)		10,639 (41.7%)	353 (69.8%)		10,128 (41.2%)	864 (58.9%)	
HEI [mean (SD)]	54.01 (13.42)	54.37 (13.44)	52.52 (13.34)	<0.001	54.08 (13.43)	50.42 (12.40)	<0.001	54.16 (13.42)	51.56 (13.23)	<0.001
DII [mean (SD)]	1.39 (1.59)	1.51 (1.58)	1.69 (1.60)	0.001	1.38 (1.59)	2.13 (1.38)	<0.001	1.36 (1.59)	1.85 (1.49)	<0.001
MED [mean (SD)]	5.70 (1.01)	5.71 (1.01)	5.57 (1.01)	<0.001	5.71 (1.01)	5.38 (0.96)	<0.001	5.71 (1.01)	5.56 (0.98)	<0.001
DASHI [mean (SD)]	3.52 (1.15)	3.56 (1.19)	3.45 (1.14)	0.049	3.52 (1.15)	3.27 (1.05)	<0.001	3.53 (1.15)	3.36 (1.12)	<0.001
HEI4 (%)				0.004			<0.001			<0.001
Q1	5,760 (25.3%)	4,778 (24.7%)	982 (28.7%)		5,597 (25.2%)	163 (30.9%)		5,334 (24.9%)	426 (31%)	
Q2	5,760 (25.2%)	4,900 (25.2%)	860 (25.2%)		5,609 (25.1%)	151 (31.8%)		5,407 (25.2%)	353 (26.1%)	
Q3	5,760 (24.7%)	4,950 (24.8%)	810 (23.8%)		5,645 (24.7%)	115 (24%)		5,439 (24.8%)	321 (23.5%)	
Q4	5,761 (24.8%)	5,018 (25.3%)	743 (22.3%)		5,701 (25%)	60 (13.3%)		5,500 (25.1%)	261 (19.4%)	
DII category (%)				<0.001			<0.001			<0.001
Q1	5,760 (28%)	5,010 (28.6%)	750 (24.7%)		5,697 (28.3%)	63 (11.8%)		5,530 (28.6%)	230 (18.2%)	
Q2	5,760 (26.1%)	4,959 (26.1%)	801 (26.3%)		5,665 (26.2%)	95 (22.9%)		5,453 (26.2%)	307 (25.8%)	
Q3	5,760 (24%)	4,911 (24.1%)	849 (23.5%)		5,610 (23.9%)	150 (29%)		5,402 (24%)	358 (23.3%)	
Q4	5,761 (21.9%)	4,767 (21.3%)	994 (25.5%)		5,580 (21.6%)	181 (36.3%)		5,295 (21.2%)	466 (32.8%)	
MEDI category (%)				<0.001			<0.001			<0.001
Q1	7,931 (34.4%)	6,611 (33.8%)	1,320 (37.3%)		7,689 (34.1%)	242 (46%)		7,359 (34%)	572 (41.1%)	
Q2	4,270 (18.3%)	3,617 (18%)	653 (19.8%)		4,173 (18.2%)	97 (20.6%)		4,018 (18.2%)	252 (18.4%)	
Q3	7,204 (31.7%)	6,233 (32.2%)	971 (29.3%)		7,104 (31.9%)	100 (23.9%)		6,834 (32%)	370 (27.5%)	
Q4	3,577 (15.7%)	3,142 (16%)	435 (13.6%)		3,531 (15.8%)	46 (9.5%)		3,416 (15.8%)	161 (13%)	
DASHI category (%)				0.22			0.006			0.011
Q1	5,761 (25%)	4,818 (24.7%)	943 (26.7%)		5,617 (25%)	144 (26.6%)		5,366 (24.8%)	395 (29.5%)	
Q2	5,760 (26%)	4,877 (26%)	883 (26%)		5,605 (25.8%)	155 (31.8%)		5,385 (25.9%)	375 (27.2%)	
Q3	5,760 (25.6%)	4,942 (25.6%)	818 (25.4%)		5,642 (25.5%)	118 (26.7%)		5,431 (25.7%)	329 (23.8%)	
Q4	5,761 (23.5%)	5,010 (23.7%)	751 (21.9%)		5,689 (23.6%)	72 (14.8%)		5,499 (23.7%)	262 (19.5%)	

BMI, body mass index; PA, physical activity; HEI, Healthy Eating Index; DII, Dietary Inflammation Index; MEDI, Mediterranean Dietary Index; DASHI, Dietary Approaches to Stop Hypertension Index; *p*, *p*-value.

### 3.2 Healthier dietary index scores are associated with a lower risk of chronic respiratory disease

A multifactorial stepwise logistic regression model was employed to examine the association between different dietary pattern index scores and the risk of chronic respiratory diseases. The stepwise logistic regression model used three covariate models. Model 1 was adjusted for demographic variables (including gender, age, race, education level, marital status, and income level), Model 2 was adjusted for demographic variables and lifestyle variables (including BMI, smoking, drinking, and PA), and Model 3 was adjusted for demographic variables, lifestyle variables, and disease covariates (hypertension and diabetes). After stepwise correction for covariates, the results of the risk-related regressions of the four dietary index scores on asthma, emphysema, and CB in the weighted sample are shown in **Tables 2**–**4**, respectively. In **Table 2**, HEI-2020, MEDI, and DASHI (both continuous and quartile categorical variables) all showed stable significant correlations with the risk of asthma. In **Table 3**, HEI-2020, MEDI (both continuous and quartile categorical variables), quartile categorical variables of DII scores, and continuous variables of DASHI scores all showed stable significant correlations with risk of emphysema. In **Table 4**, only HEI-2020 (both continuous and quartile categorical variables) showed stable significant correlations with risk of CB [OR of HEI-2020 continuous:0.99 (0.983,0.997), *p* < 0.001; OR of HEI-2020 Q4 vs. Q1: 0.711 (0.526,0.961), *p* = 0.027].

**Table 2:** Relationship between different dietary indices and asthma among adults aged 20 years or older.

Variable	OR (95% CI) simple without weighted			*p*	OR (95% CI) simple with weighted			*p*
	Model 1	Model 2	Model 3		Model 1	Model 2	Model 3	
HEI-2020 continuous	0.991 (0.988,0.994)	0.993 (0.989,0.996)	0.993 (0.989,0.996)	<0.001	0.992 (0.987,0.996)	0.993 (0.988,0.997)	0.993 (0.988,0.997)	<0.001
HEI-2020 category (ref Q1)								
Q2	0.891 (0.806,0.986)	0.844 (0.761,0.936)	0.773 (0.693,0.862)	<0.001	0.884 (0.755,1.034)	0.851 (0.739,0.981)	0.782 (0.66,0.927)	<0.001
Q3	0.944 (0.842,1.058)	0.915 (0.811,1.032)	0.818 (0.715,0.935)	0.003	0.943 (0.777,1.144)	0.888 (0.768,1.025)	0.824 (0.694,0.977)	0.026
Q4	0.945 (0.843,1.059)	0.91 (0.806,1.027)	0.817 (0.714,0.934)	0.003	0.945 (0.778,1.147)	0.887 (0.767,1.025)	0.823 (0.693,0.978)	0.027
DII continuous	1.041 (1.015,1.068)	1.044 (1.012,1.076)	1.042 (1.01,1.075)	0.009	1.032 (0.99,1.076)	1.045 (0.999,1.093)	1.044 (0.998,1.092)	0.006
DII category (ref Q1)								
Q2	1.031 (0.925,1.149)	1.048 (0.94,1.168)	1.202 (1.078,1.34)	0.001	1.102 (0.949,1.279)	1.018 (0.87,1.192)	1.171 (0.993,1.38)	0.060
Q3	1.019 (0.892,1.165)	1.023 (0.896,1.169)	1.154 (1.01,1.318)	0.035	1.122 (0.938,1.342)	1.032 (0.869,1.224)	1.161 (0.967,1.394)	0.108
Q4	1.015 (0.888,1.161)	1.015 (0.889,1.16)	1.146 (1.004,1.31)	0.044	1.12 (0.936,1.341)	1.026 (0.865,1.218)	1.157 (0.963,1.39)	0.117
MEDI continuous	0.89 (0.856,0.926)	0.913 (0.871,0.957)	0.913 (0.871,0.958)	<0.001	0.918 (0.872,0.966)	0.934 (0.881,0.991)	0.935 (0.881,0.992)	0.026
MEDI category (ref Q1)								
Q2	0.935 (0.842,1.036)	0.827 (0.753,0.907)	0.74 (0.654,0.835)	<0.001	1.003 (0.877,1.148)	0.853 (0.748,0.972)	0.786 (0.665,0.928)	0.005
Q3	0.977 (0.867,1.099)	0.892 (0.8,0.995)	0.755 (0.643,0.884)	0.001	1.044 (0.892,1.222)	0.9 (0.78,1.039)	0.79 (0.647,0.964)	0.021
Q4	0.969 (0.86,1.091)	0.891 (0.798,0.994)	0.758 (0.645,0.887)	0.001	1.037 (0.886,1.214)	0.899 (0.78,1.037)	0.793 (0.65,0.967)	0.022
DASHI continuous	0.938 (0.908,0.969)	0.95 (0.913,0.988)	0.949 (0.913,0.988)	0.010	0.947 (0.899,0.999)	0.941 (0.886,1)	0.94 (0.885,0.999)	0.045
DASHI category (ref Q1)								
Q2	0.931 (0.841,1.03)	0.87 (0.785,0.965)	0.807 (0.725,0.899)	<0.001	0.91 (0.786,1.053)	0.904 (0.795,1.028)	0.842 (0.71,0.997)	0.046
Q3	0.942 (0.839,1.057)	0.877 (0.778,0.99)	0.826 (0.725,0.94)	0.004	0.871 (0.733,1.035)	0.845 (0.725,0.985)	0.813 (0.672,0.985)	0.034
Q4	0.937 (0.835,1.052)	0.877 (0.777,0.989)	0.824 (0.723,0.937)	0.003	0.87 (0.732,1.035)	0.844 (0.724,0.984)	0.811 (0.67,0.981)	0.031

Model 1: sex, age group, race, income, education, marital status.

Model 2: sex, age group, race, income, education, marital status, BMI, smoking, drinking, physical activity.

Model 3: sex, age group, race, income, education, marital status, BMI, smoking, drinking, physical activity, diabetes, hypertension.

HEI, Healthy Eating Index; DII, Dietary Inflammation Index; MEDI, Mediterranean Dietary Index; DASHI, Dietary Approaches to Stop Hypertension Index; *p*, *p*-value for model 3.

**Table 3:** Relationship between different dietary indices and emphysema among adults aged 20 years or older.

Variable	OR (95% CI) simple without weighted			*p*	OR (95% CI) simple with weighted			*p*
	Model 1	Model 2	Model 3		Model 1	Model 2	Model 3	
HEI-2020 continuous	0.973 (0.965,0.98)	0.982 (0.974,0.99)	0.982 (0.974,0.99)	<0.001	0.976 (0.967,0.985)	0.988 (0.977,1)	0.993 (0.988,0.997)	<0.001
HEI-2020 category (ref Q1)								
Q2	0.902 (0.714,1.138)	0.666 (0.517,0.856)	0.346 (0.251,0.47)	<0.001	0.951 (0.712,1.27)	0.699 (0.519,0.941)	0.389 (0.26,0.584)	<0.001
Q3	1.027 (0.799,1.318)	0.828 (0.627,1.088)	0.46 (0.317,0.656)	<0.001	1.122 (0.801,1.572)	0.959 (0.674,1.365)	0.577 (0.354,0.942)	0.028
Q4	1.023 (0.796,1.314)	0.82 (0.621,1.078)	0.457 (0.314,0.651)	<0.001	1.123 (0.801,1.574)	0.96 (0.676,1.362)	0.572 (0.353,0.927)	0.024
DII continuous	1.257 (1.175,1.347)	1.169 (1.084,1.264)	1.042 (1.01,1.075)	0.009	1.308 (1.197,1.429)	1.191 (1.068,1.328)	1.044 (0.998,1.092)	0.062
DII category (ref Q1)								
Q2	1.359 (0.983,1.891)	2.118 (1.567,2.895)	2.247 (1.666,3.068)	<0.001	1.922 (1.258,2.936)	2.592 (1.578,4.257)	3.002 (1.972,4.571)	<0.001
Q3	1.232 (0.855,1.79)	1.762 (1.256,2.507)	1.721 (1.229,2.447)	0.002	1.553 (1.018,2.371)	1.985 (1.16,3.395)	1.99 (1.257,3.151)	0.003
Q4	1.236 (0.858,1.795)	1.745 (1.243,2.484)	1.716 (1.225,2.441)	0.002	1.549 (1.01,2.375)	1.973 (1.147,3.395)	1.985 (1.25,3.154)	0.004
MEDI continuous	0.68 (0.616,0.75)	0.75 (0.671,0.838)	0.913 (0.871,0.958)	<0.001	0.711 (0.64,0.79)	0.809 (0.706,0.928)	0.935 (0.881,0.992)	0.026
MEDI category (ref Q1)								
Q2	0.725 (0.564,0.925)	0.439 (0.342,0.559)	0.4 (0.284,0.552)	<0.001	0.827 (0.604,1.132)	0.53 (0.393,0.713)	0.415 (0.265,0.648)	<0.001
Q3	0.839 (0.641,1.088)	0.565 (0.429,0.738)	0.44 (0.282,0.661)	<0.001	0.956 (0.669,1.367)	0.799 (0.553,1.153)	0.432 (0.258,0.723)	0.001
Q4	0.829 (0.634,1.076)	0.563 (0.428,0.736)	0.442 (0.284,0.665)	<0.001	0.95 (0.663,1.362)	0.797 (0.552,1.151)	0.435 (0.26,0.727)	0.001
DASHI continuous	0.823 (0.757,0.894)	0.878 (0.799,0.963)	0.949 (0.913,0.988)	0.010	0.844 (0.755,0.942)	0.932 (0.824,1.053)	0.94 (0.885,0.999)	0.045
DASHI category (ref Q1)								
Q2	1.142 (0.901,1.449)	0.9 (0.697,1.16)	0.534 (0.395,0.717)	<0.001	1.244 (0.905,1.711)	1.097 (0.766,1.571)	0.616 (0.389,0.975)	0.038
Q3	1.21 (0.937,1.562)	0.979 (0.739,1.293)	0.654 (0.466,0.908)	0.012	1.394 (1,1.945)	1.229 (0.816,1.85)	0.895 (0.558,1.436)	0.642
Q4	1.206 (0.934,1.557)	0.976 (0.737,1.29)	0.651 (0.464,0.904)	0.012	1.391 (0.998,1.938)	1.227 (0.812,1.854)	0.887 (0.556,1.417)	0.611

Model 1: sex, age group, race, income, education, marital status.

Model 2: sex, age group, race, income, education, marital status, BMI, smoking, drinking, physical activity.

Model 3: sex, age group, race, income, education, marital status, BMI, smoking, drinking, physical activity, diabetes, hypertension.

HEI, Healthy Eating Index; DII, Dietary Inflammation Index; MEDI, Mediterranean Dietary Index; DASHI, Dietary Approaches to Stop Hypertension Index; *p*, *p*-value for model 3.

**Table 4:** Relationship between different dietary indices and CB among adults aged 20 years or older.

Variable	OR (95% CI) simple without weighted			*p*	OR (95% CI) simple with weighted			*p*
	Model 1	Model 2	Model 3		Model 1	Model 2	Model 3	
HEI-2020 continuous	0.984 (0.979,0.988)	0.989 (0.984,0.995)	0.989 (0.984,0.994)	<0.001	0.983 (0.977,0.99)	0.99 (0.983,0.997)	0.99 (0.983,0.997)	0.005
HEI-2020 category (ref Q1)								
Q2	0.811 (0.699,0.942)	0.727 (0.622,0.848)	0.579 (0.489,0.685)	<0.001	0.814 (0.673,0.984)	0.727 (0.587,0.899)	0.588 (0.453,0.763)	<0.001
Q3	0.847 (0.718,0.998)	0.859 (0.721,1.021)	0.681 (0.555,0.832)	<0.001	0.829 (0.672,1.024)	0.881 (0.698,1.112)	0.718 (0.532,0.968)	0.030
Q4	0.846 (0.717,0.997)	0.849 (0.712,1.01)	0.678 (0.553,0.828)	<0.001	0.832 (0.673,1.028)	0.879 (0.696,1.111)	0.711 (0.526,0.961)	0.027
DII continuous	1.114 (1.071,1.159)	1.077 (1.028,1.128)	1.074 (1.025,1.126)	0.003	1.125 (1.059,1.196)	1.087 (1.011,1.167)	1.085 (1.009,1.166)	0.027
DII category (ref Q1)								
Q2	1.229 (1.03,1.469)	1.318 (1.107,1.571)	1.545 (1.303,1.837)	<0.001	1.396 (1.059,1.840)	1.238 (0.936,1.637)	1.691 (1.263,2.264)	<0.001
Q3	1.136 (0.918,1.409)	1.237 (1.006,1.525)	1.312 (1.07,1.615)	0.010	1.304 (0.940,1.810)	1.13 (0.814,1.569)	1.413 (1.002,1.993)	0.048
Q4	1.132 (0.914,1.405)	1.218 (0.991,1.503)	1.300 (1.060,1.600)	0.012	1.299 (0.939,1.797)	1.114 (0.802,1.548)	1.404 (0.994,1.983)	0.053
MEDI continuous	0.832 (0.784,0.882)	0.899 (0.839,0.964)	0.897 (0.837,0.962)	0.002	0.856 (0.794,0.923)	0.941 (0.868,1.021)	0.941 (0.867,1.021)	0.139
MEDI category (ref Q1)								
Q2	0.807 (0.689,0.942)	0.697 (0.606,0.802)	0.598 (0.494,0.721)	<0.001	0.83 (0.672,1.026)	0.7 (0.577,0.849)	0.65 (0.495,0.854)	0.002
Q3	0.849 (0.712,1.01)	0.832 (0.708,0.975)	0.658 (0.513,0.835)	0.001	0.885 (0.703,1.113)	0.859 (0.682,1.082)	0.785 (0.562,1.097)	0.154
Q4	0.831 (0.696,0.989)	0.828 (0.704,0.971)	0.661 (0.516,0.839)	0.001	0.863 (0.685,1.087)	0.852 (0.678,1.07)	0.791 (0.566,1.107)	0.168
DASHI continuous	0.879 (0.837,0.924)	0.917 (0.865,0.971)	0.916 (0.864,0.97)	0.003	0.871 (0.81,0.938)	0.929 (0.855,1.01)	0.926 (0.852,1.006)	0.069
DASHI category (ref Q1)								
Q2	0.946 (0.815,1.098)	0.837 (0.717,0.976)	0.647 (0.547,0.764)	<0.001	0.872 (0.703,1.081)	0.779 (0.63,0.965)	0.661 (0.521,0.839)	<0.001
Q3	0.961 (0.815,1.133)	0.873 (0.732,1.04)	0.73 (0.6,0.885)	0.001	0.937 (0.746,1.178)	0.811 (0.64,1.028)	0.817 (0.627,1.064)	0.131
Q4	0.952 (0.807,1.123)	0.87 (0.729,1.037)	0.725 (0.596,0.879)	0.001	0.934 (0.743,1.173)	0.808 (0.634,1.03)	0.806 (0.62,1.049)	0.107

Model 1: sex, age group, race, income, education, marital status.

Model 2: sex, age group, race, income, education, marital status, BMI, smoking, drinking, physical activity.

Model 3: sex, age group, race, income, education, marital status, BMI, smoking, drinking, physical activity, diabetes, hypertension.

HEI, Healthy Eating Index; DII, Dietary Inflammation Index; MEDI, Mediterranean Dietary Index; DASHI, Dietary Approaches to Stop Hypertension Index; *p*, *p*-value for model 3.

### 3.3 Dose–response relationship between dietary index scores and risk of chronic respiratory disease

**Figure 2:**
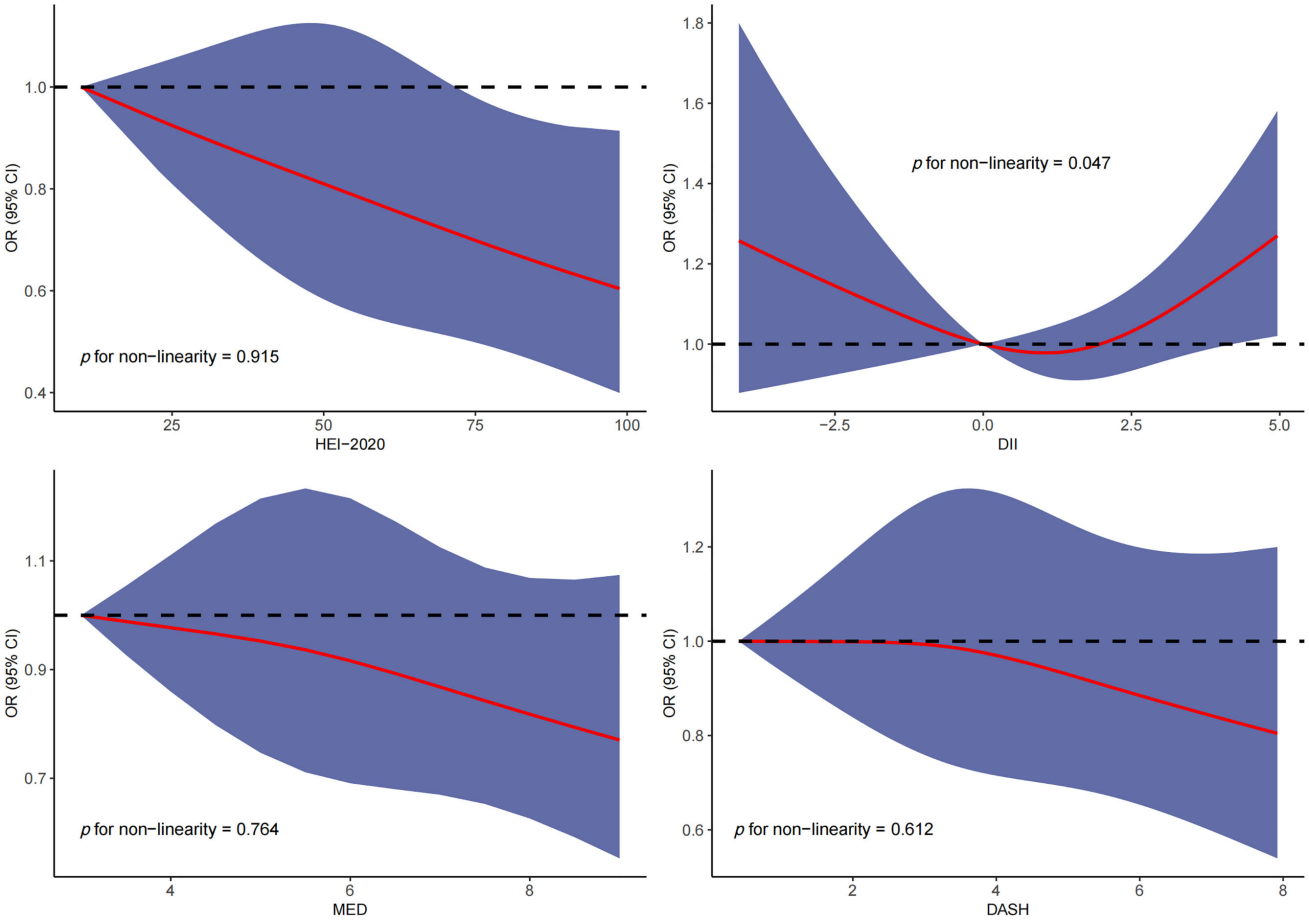
Dose–response relationship results between four dietary indices (HEI-2020, DII, MEDI, and DASHI) and OR ratios in the RCS-asthma model.

**Figure 3:**
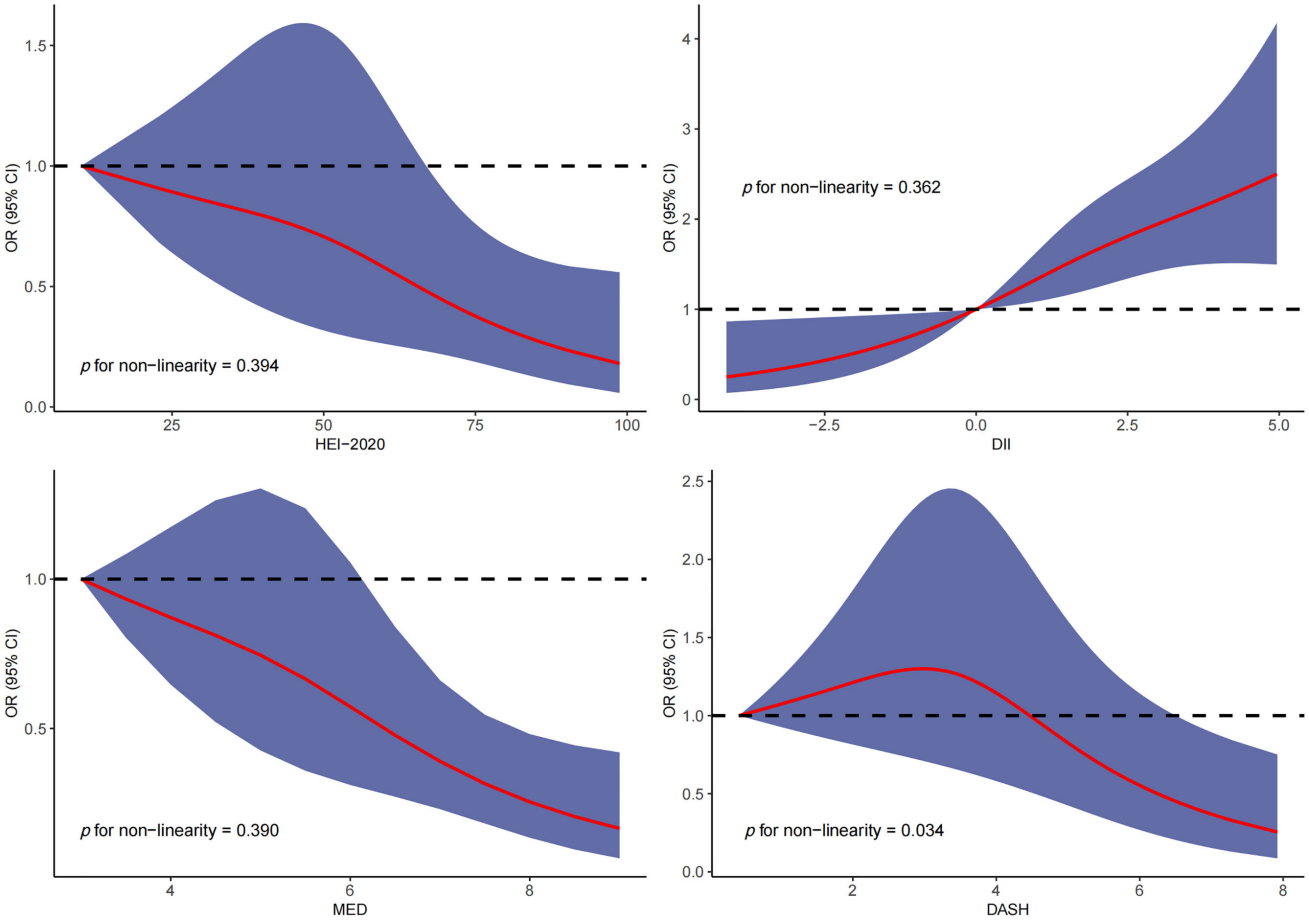
Dose–response relationship results between four dietary indices (HEI-2020, DII, MEDI, and DASHI) and OR ratios in the RCS-emphysema model.

**Figure 4:**
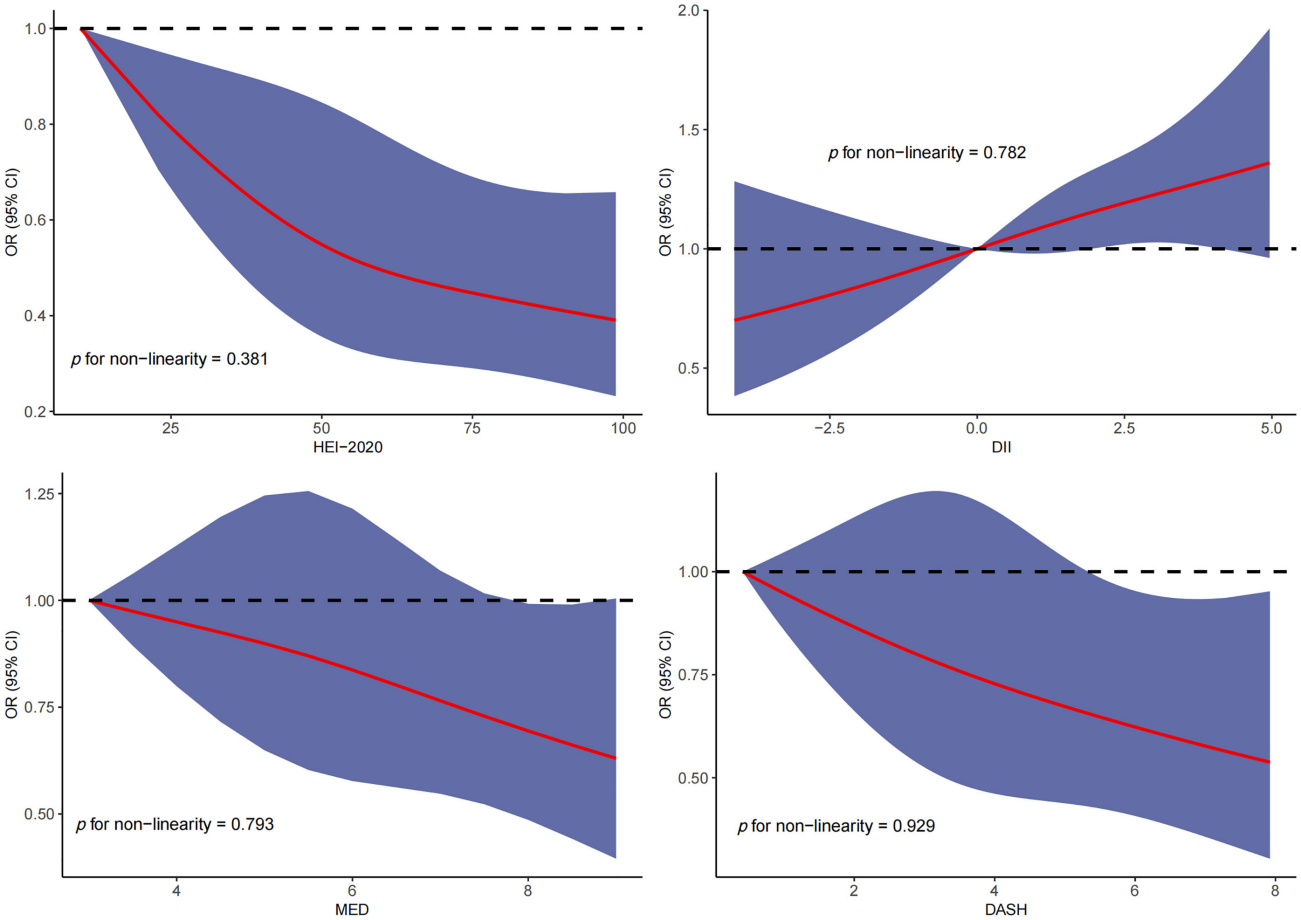
Dose–response relationship results between four dietary indices (HEI-2020, DII, MEDI, and DASHI) and OR ratios in the RCS-chronic bronchitis (CB) model.

RCS was used to explore the dose–response relationship between different dietary pattern index scores and the risk of three chronic respiratory diseases. The results of RCS demonstrated a significant dose–response relationship between HEI-2020 and the risk of asthma without concomitant nonlinear relationships (**Figure 2**). The results of RCS also demonstrated a significant dose–response relationship between all four dietary indices and the risk of emphysema without nonlinear relationships in **Figure 3**. Similarly, the four dietary index scores had a significant dose–response relationship with the risk of CB in **Figure 4**. There was no nonlinear relationship between them.

### 3.4 Mixed effects of 13 dietary components on chronic respiratory disease

**Table 5** shows the mixed effects of the dietary components of the four kinds of dietary index on the risk of chronic respiratory disease in the model of WQS.

**Table 5:** Relationship between the mixed effects of the dietary components of the HEI-2020 and chronic respiratory disease among adults aged 20 years or older.

Disease outcome	HEI-2020		DII		MEDI		DASHI	
	OR (95% CI)	*p*	OR (95% CI)	*p*	OR (95% CI)	*p*	OR (95% CI)	*p*
Asthma								
Model 1	0.953 (0.933,0.973)	<0.001	1.335 (1.07,1.667)	0.011	0.558 (0.411,0.759)	<0.001	0.821 (0.759,0.888)	<0.001
Model 2	0.949 (0.920,0.979)	0.001	1.122 (0.962,1.309)	0.142	0.552 (0.378,0.804)	0.002	0.836 (0.748,0.935)	0.002
Model 3	0.947 (0.919,0.976)	<0.001	1.121 (0.944,1.331)	0.192	0.55 (0.376,0.804)	0.002	0.829 (0.742,0.926)	0.001
Emphysema								
Model 1	0.903 (0.855,0.954)	<0.001	2.700 (1.699,4.290)	<0.001	0.274 (0.143,0.526)	<0.001	0.674 (0.53,0.858)	0.001
Model 2	0.859 (0.802,0.921)	<0.001	2.013 (1.178,3.441)	0.011	0.219 (0.108,0.443)	<0.001	0.688 (0.569,0.832)	<0.001
Model 3	0.86 (0.803,0.921)	<0.001	2.07 (1.209,3.546)	0.008	0.24 (0.122,0.473)	<0.001	0.691 (0.574,0.832)	<0.001
CB								
Model 1	0.905 (0.875,0.937)	<0.001	2.119 (1.537,2.921)	<0.001	0.475 (0.300,0.752)	0.001	0.708 (0.615,0.815)	<0.001
Model 2	0.895 (0.853,0.939)	<0.001	1.465 (1.043,2.059)	0.028	0.574 (0.340,0.971)	0.038	0.789 (0.669,0.932)	0.005
Model 3	0.898 (0.857,0.94)	<0.001	1.454 (1.037,2.04)	0.030	0.585 (0.346,0.989)	0.046	0.777 (0.660,0.915)	0.003

Model 1: sex, age group, race, income, education, marital status.

Model 2: sex, age group, race, income, education, marital status, BMI, smoking, drinking, physical activity.

Model 3: sex, age group, race, income, education, marital status, BMI, smoking, drinking, physical activity, diabetes, hypertension.

HEI, Healthy Eating Index; DII, Dietary Inflammation Index; MEDI, Mediterranean Dietary Index; DASHI, Dietary Approaches to Stop Hypertension Index.

After stepwise inclusion of covariates, HEI-2020, MEDI, and DASHI exhibited significant health mixing effects on the risk of asthma in the WQS-asthma model [HEI-2020 OR: 0.947 (0.919,0.976); MEDI OR: 0.55 (0.376,0.804); DASHI OR: 0.829 (0.742,0.926)]. HEI-2020, DII, MEDI, and DASHI have shown significant mixing effects on the risk of emphysema in the WQS-emphysema model [HEI-2020 OR: 0.86 (0.803,0.921); DII OR: 2.07 (1.209,3.546); MEDI OR: 0.24 (0.122,0.473); DASHI OR: 0.691 (0.574,0.832)]. In the WQS-CB model, HEI-2020, DII, MEDI, and DASHI have shown significant mixing effects on the risk of CB [HEI-2020 OR: 0.898 (0.857,0.94); DII OR: 1.454 (1.037,2.04); MEDI OR: 0.585 (0.346,0.989); DASHI OR: 0.777 (0.660,0.915)].

**Figure 5:**
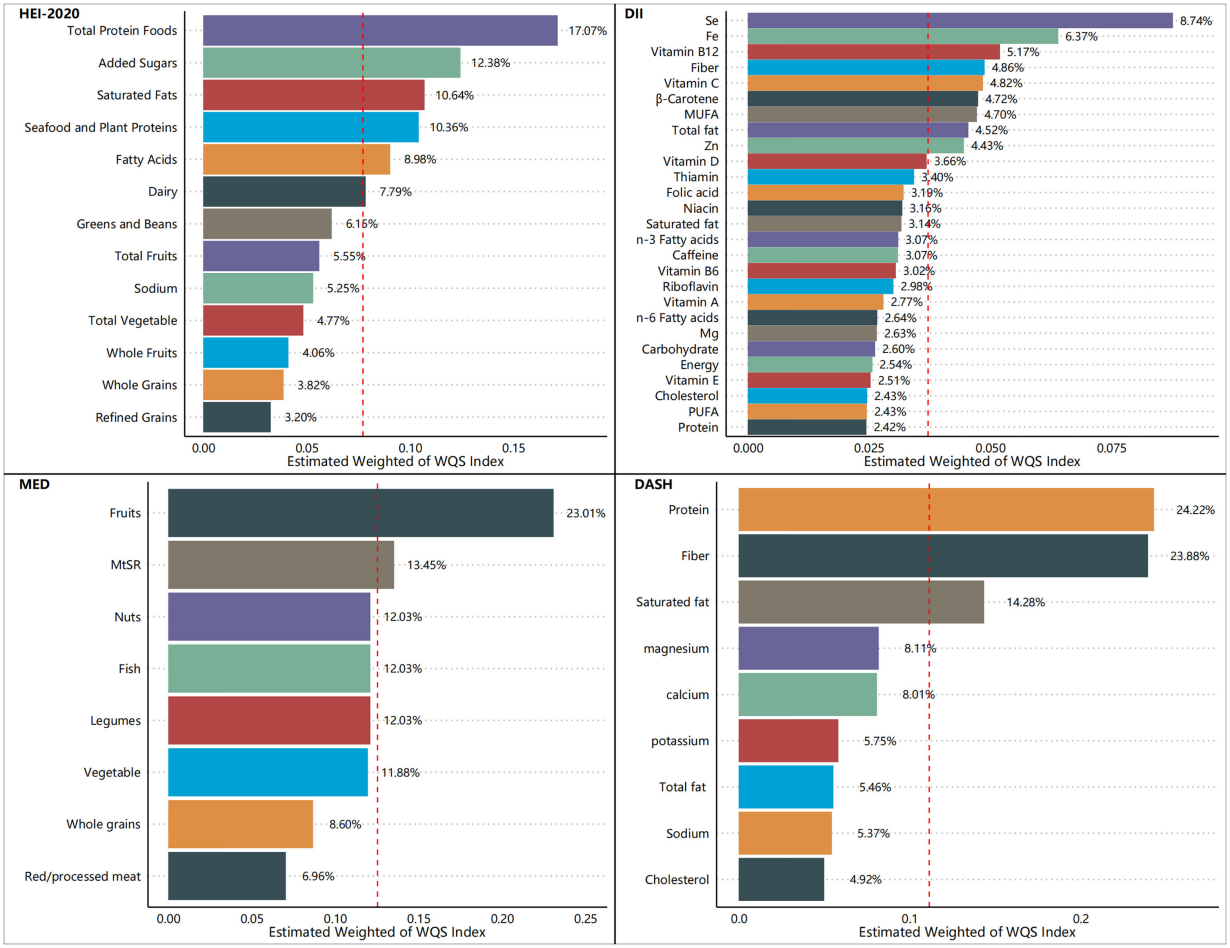
Contribution weights of dietary components in the WQS-asthma model.

**Figure 6:**
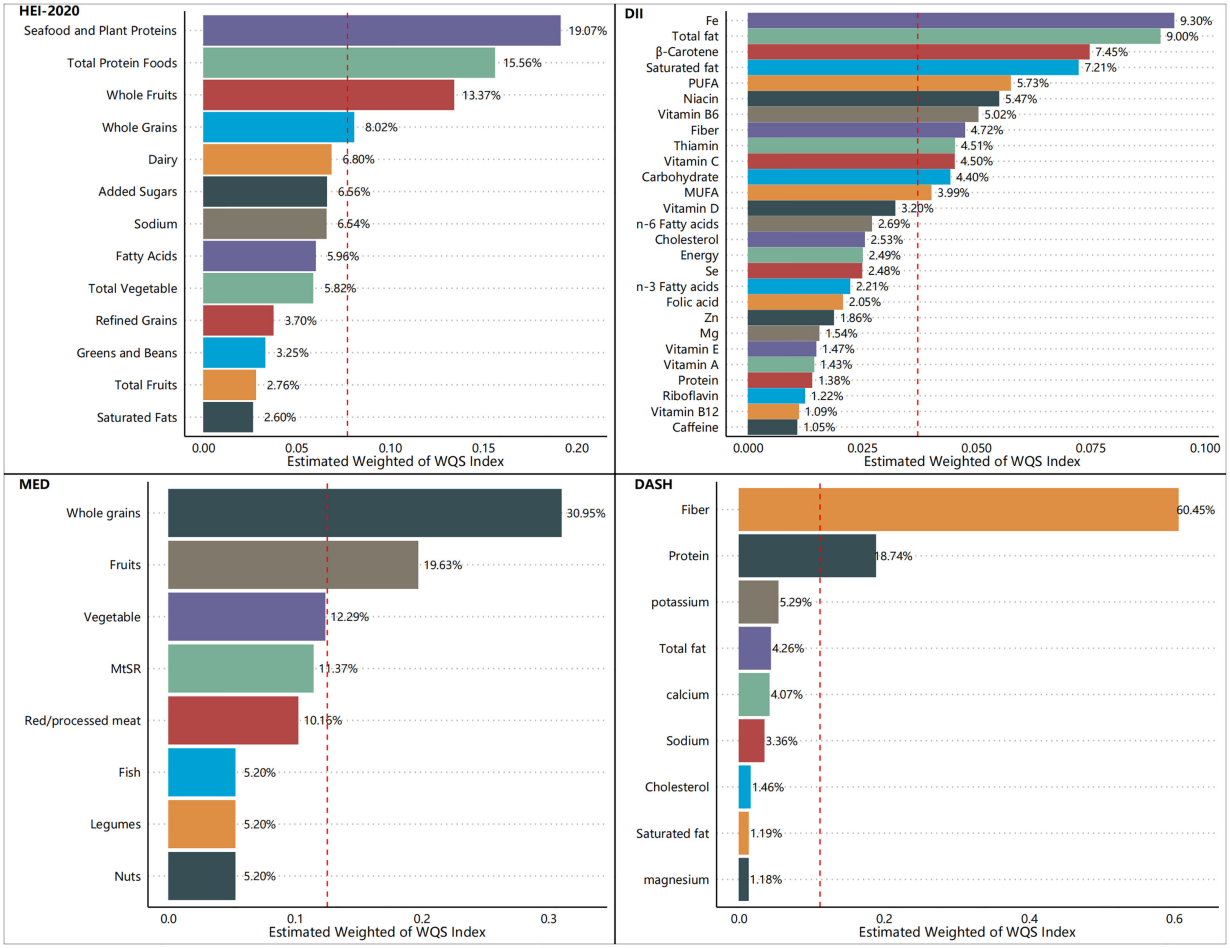
Contribution weights of dietary components in the WQS-emphysema model.

**Figure 7:**
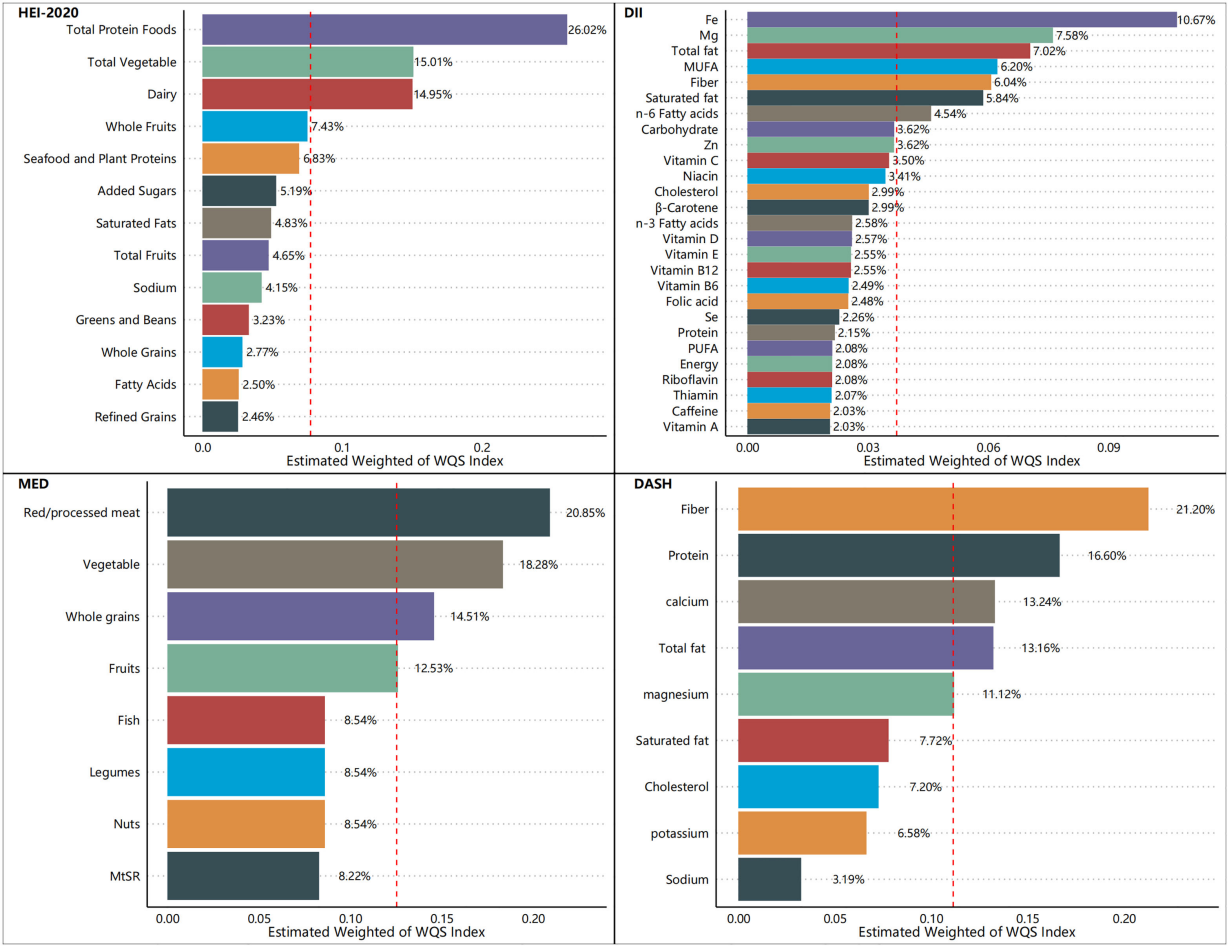
Contribution weights of dietary components in the WQS-chronic bronchitis (CB) model.

**Figures 5**–**7** show the results of the mixed effects of dietary components from various dietary indices on the risk of CRD within the WQS model. In the WQS-asthma model, the most contributing dietary components in HEI-2020, DII, MEDI, and DASHI were total protein foods (17.07%), Se (8.74%), fruits (23.01%), and proteins (24.22%), respectively (**Figure 5**). In the WQS of the emphysema model, the dietary components that contributed the most to HEI-2020, DII, MEDI, and DASHI were seafood and plant protein (19.07%), Fe (9.30%), whole grains (30.95%), and dietary fiber (60.45%), respectively (**Figure 6**). In the WQS-CB model, the dietary components that contributed the most to HEI-2020, DII, MEDI, and DASHI were seafood and plant protein (26.02%), Fe (10.67%), red/processed meat (20.85%), and dietary fiber (21.20%), respectively (**Figure 7**).

### 3.5 Subgroup analysis of the correlation between dietary index scores and chronic respiratory diseases

We also performed subgroup analyses by gender and found that the relationship between dietary index scores and CRD risk varied by gender. In comparison to female patients, the significant relationship between high dietary quality and the low risk of CRD in male patients was more stable across dietary scores and across population proportions (**Supplementary Tables 2** **−** **4**).

The RCS was also used to explore the dose–response relationship between dietary index scores and risk of chronic respiratory disease by gender (**Supplementary Figures 1**−**6**). It was found that the dose–response relationship between the continuous dietary quality scores and the risk of CRD was more significant in men compared to women, while the dose–response relationship between the four dietary quality scores and the risk of emphysema was the most significant and stable in men, and there was no nonlinear relationship between them.

## 4 Discussion

Our research encompassed a sample of 23,042 U.S. adults, which, after appropriate weighting, is representative of approximately 160 million non-institutionalized U.S. adults. Three positive rating indicators (HEI-2020, MEDI, and DASHI) and one negative rating indicator (DII) were used together to assess the relationship between dietary quality and respiratory health. Higher healthy diet scores (HEI-2020, MEDI, and DASHI) and lower unhealthy diet scores (DII) were also found to be associated with a lower risk of respiratory disease in the results. This suggests a strong relationship between dietary quality and respiratory health, as well as a consistent relationship between different dietary scores and respiratory health, and the robustness of the results of this study. Moreover, this correlation appears to be robust, holding steady across variations in sample demographics and differing definitions of dietary quality. Among the dietary indices evaluated, the HEI-2020 consistently demonstrated the most pronounced health benefits, with both its continuous and quartile scores significantly correlating with a lower risk of the aforementioned respiratory diseases in both unweighted and weighted samples. The restricted cubic spline (RCS) analysis revealed significant inverse relationships between the three health-promoting dietary indices (HEI-2020, MEDI, and DASHI) and respiratory disease risk, while a significant positive association was observed with the DII. These findings were corroborated by the outcomes of logistic regression analyses, which further substantiated the stability and reliability of our results. The Weighted Quantile Sum (WQS) model also identified a salutary interaction between the three dietary indices (HEI-2020, MEDI, and DASHI) and the risk of respiratory disease, in contrast to an adverse interaction observed between the DII score and respiratory disease risk. Additionally, we conducted an in-depth analysis to determine the individual contributions of various dietary components to the risk of respiratory disease, considering their proportion relative to the total dietary mass. When the dietary index was decomposed into its constituent food elements, the specific dietary components of the DII were examined for their influence on respiratory disease risk. In the context of asthma risk, the food groups that predominantly influenced the scores of the dietary indices (HEI-2020, DII, MEDI, and DASHI) were total protein foods (17.07%), selenium (Se, 8.74%), fruits (23.01%), and proteins (24.22%), respectively. Regarding the risk of emphysema, the food groups that had the most significant impact on the dietary indices scores were seafood and plant protein (19.07%), iron (Fe, 9.30%), whole grains (30.95%), and dietary fiber (60.45%), respectively. For the risk of CB, the food groups that were most influential to the scores of the dietary indices were seafood and plant protein (26.02%), iron (Fe, 10.67%), red/processed meat (20.85%), and dietary fiber (21.20%), respectively. These findings underscore the substantial health potential of proteins, particularly high-quality sources such as plant and seafood proteins, minerals, fruits, and vegetables, which may be beneficial in mitigating the risk of respiratory diseases.

In 2014, the European Respiratory Society released a statement highlighting the importance of nutritional assessment and treatment for COPD (45). The role of nutrition in managing chronic respiratory diseases has gained widespread recognition in recent years (46–48). Western dietary patterns, notably marked by an elevated intake of preserved and processed meats, are correlated with a heightened risk of chronic respiratory diseases. This correlation could be due to the inflammatory consequences of excessive sugar, salt, and nitrite consumption from processed foods (49, 50). Furthermore, an array of studies have reported the positive impact of the DII, DASH, and the Mediterranean diet on respiratory health (51–55). In their study on the effects of plant-based diets, Alwarith et al. discovered that such diets were linked to a decrease in asthma attacks and improvement in symptoms. These effects may be attributed to the modulation of cytokine release, mitigation of free radical damage, and modulation of immune responses, all of which play a role in the onset and progression of asthma (56). These findings are consistent with the results of our study.

Proteases and antiproteases are secreted by the respiratory epithelium and are involved in the immune homeostatic balance of the respiratory tract. Alterations in protease/antiprotease balance can lead to the development of lung diseases such as emphysema or COPD (57). Supplementation of the dietary route with nutritive antioxidants, such as flavonoids, may enhance respiratory mucosal responses and/or prevent infections, reducing the risk of respiratory disease development (58). This also demonstrates the benefits of fruit and vegetable intake for respiratory health. Malnutrition has been found to be associated with poor quality of survival in COPD patients in different observational studies (59, 60). Nutritional therapy has notably demonstrated its efficacy in sustaining and enhancing muscle strength and exercise tolerance among malnourished COPD patients (61). Studies have pinpointed low body weight and diminished fat-free mass (FFM) as ominous prognostic indicators in COPD patients, with evidence suggesting that a diet rich in high-quality protein or essential amino acid supplementation can bolster the fat-free body weight index and elevate arterial oxygen saturation levels (62, 63). A study delving into protein absorption and utilization among COPD patients has uncovered that inadequate protein intake, systemic inflammation, and hypertension are predictors of a reduced postabsorptive protein balance, which correlates with diminished daily physical performance (64). Individuals suffering from COPD frequently present with clinical malnutrition, particularly protein deficiency, which can precipitate dyspnea or skeletal muscle dysfunction (65–67). The significance of minerals, such as selenium (Se) and iron (Fe), in respiratory health has been underscored by recent research (68, 69), aligning with findings that a deficiency in these minerals in the DII can heighten the inflammatory potential of one’s diet. Concurrently, two extensive cohort studies in the U.S. have correlated a higher consumption of marine fish with a decreased risk of developing COPD (70). Moreover, additional research has indicated that the intake of whole grains or dietary fiber may also mitigate the risk of chronic respiratory diseases (65, 71, 72), potentially due to the fiber’s ability to modulate the gut microbiota and augment short-chain fatty acid production, which, in turn, regulates neutrophil activity and mitigates allergic responses (73). Collectively, these studies underscore the advantageous impact of protein, mineral, fruit, and vegetable consumption on the preservation of respiratory health.

This study has certain strengths; most notably, it utilized a multi-model sensitivity analysis design: (1) four dietary score approaches (HEI-2020, DII, MEDI, and DASHI) were used to define dietary quality; (2) the study population samples were weighted and transformed into different population proportions, unweighted and weighted samples, and subgroups were analyzed by gender; and (3) dietary index scores were also classified into categorical continuous variables and quartiles of the variables, to make the results more realistic and reliable. The second is the use of a large U.S. Nutrition and Health Survey database, which is reliable and representative, and lastly, the use of the latest version of the HEI, HEI-2020, which is based on DGA 2020–2025, which is representative of the U.S. population’s dietary intake, and dietary indices of three mainstream nutritional studies.

In addition, there are shortcomings in this study, the largest being that this was a cross-sectional study and could not validate the causal link between dietary quality and the risk of chronic respiratory disease. Secondly, unknown confounders and potential model overfitting problems are also a shortcoming of this study. Third, the inability to standardize the dietary index components is also one of the shortcomings of this study.

The study found that high dietary quality scores were associated with low chronic respiratory risk, suggesting that improving the quality of the diet itself by following different healthy dietary patterns, especially the dietary patterns represented by the HEIs from the DGA 2020–2025 recommendations, could help prevent the occurrence and exacerbation of chronic respiratory diseases.

## 5 Conclusion

Low-quality diets, lacking in high-quality protein, minerals, and fruits and vegetables rich in dietary fiber, are associated with a higher risk of chronic respiratory disease, regardless of the dietary index used to measure diet quality.

## Data Availability

Publicly available datasets were analyzed in this study. This data can be found here: The NHANES dataset is publicly available online, accessible at cdc.gov/nchs/nhanes/index.htm.
