# Supplementary material for: Association of dietary patterns with chronic respiratory health among U.S. adults
Source: Front Immunol. 2024 Dec 6;15:1457860. doi: 10.3389/fimmu.2024.1457860 (PMC11659122; doi:10.3389/fimmu.2024.1457860)
Supplement: Supplementary file 1 [file DataSheet1.docx]

# Supplementary Materials

# Diet quality

**HEI-2020** is to assess the adherence to DGA 2020-2025, which consists of 13 components^[1]^: the adequacy components (total vegetables, greens and beans, total fruits, whole fruits, whole grains, dairy, total protein foods, seafood, and plant proteins, fatty acids) and the moderation components (sodium, refined grains, saturated fats, added sugars). Each component was given different weights and different maximum scores. HEI-2020 scores ranged from 0–100, with higher HEI scores reflecting better diet quality. **The Dietary Inflammatory Index (DII)** was developed to reflect the inflammatory potential of diets on the organism, and due to the limitations of the data related to the NHANES database utilized, a total of 28 dietary components were included in the present study for DII calculations such as vitamin A, vitamin C, vitamin D, group B vitamins, proteins, and dietary fiber, among others. unsaturated fatty acids, and dietary fiber, among others. The DII was calculated by subtracting the world average from the actual intake and dividing by the standard deviation to obtain the z-value. To control for bias, this was converted to percentile scores and the percentile scores were doubled and subtracted by 1.0 to center on 0. These centered scores were then multiplied by the Inflammatory Effects Score, and all food parameters were summed. For more details on the DII calculation, please refer to the original development research ^[2]^. A negative DII score indicates a high dietary anti-inflammatory potential; a score of 0 indicates a diet with no anti-inflammatory potential; a positive DII score indicates a high dietary pro-inflammatory potential. **The MEDI score** was adapted from the Mediterranean diet scale by Trichopoulou et al^[3]^. A total of 8 dietary components of MEDI include vegetables, fruits, nuts, whole grains, legumes, fish, monounsaturated to saturated fat ratio (MtSR), red and processed meats. A higher MEDI score indicates reflecting better diet quality^[4]^. **The DASH index** (DASHI) was used to assess the consistency of the DASH dietary patterns, and we chose the version of the DASHI that has been developed and validated by Mellen PB et al^[5]^. It contains 9 dietary components (total fat, saturated fat, protein, fiber, cholesterol, calcium, magnesium, sodium, and potassium). Higher DASHI scores reflecting better diet quality.

**Supplement Table 1 Characteristics of the four dietary indices**

| **Characteristics** |  |  |  |  |
| --- | --- | --- | --- | --- |
|  | **HEI-2020** | **DII** | **MEDI** | **DASHI** |
| Scores | 0-100 | (-,+) | 0-8 | 0-9 |
| Association with health | negative | postive | negative | negative |
| Compounds | 13 | 28 | 8 | 9 |
| Basis of development | DAG^[1]^ | Dietary Inflammation Study^[2]^ | MED patterns^[3]^ | DASH patterns^[5]^ |

**HEI**: Healthy Eating Index; **DII**: Dietary Inflammation Index; **MEDI**: Mediterranean Diet Index; **DASHI**: Dietary Approaches to Stop Hypertension Index

**Supplement Table 2** Relationship between different dietary indices and asthma among subgroup by gender.

| **Variable** | **OR (95%CI) simple without weighted** | | | | **OR (95%CI) simple with weighted** | | | |
| --- | --- | --- | --- | --- | --- | --- | --- | --- |
|  | **Male** | ***P*** | **Female** | ***P*** | **Male** | ***P*** | **Female** | ***P*** |
| **HEI-2020 continous** | 0.983(0.977,0.99) | <0.001 | 0.99(0.983,0.997) | 0.007 | 0.993(0.986,0.999) | 0.038 | 0.992(0.986,0.999) | 0.018 |
| **HEI-2020 Category(ref Q1)** |  |  |  |  |  |  |  |  |
| **Q2** | 0.953(0.806,1.127) | 0.574 | 0.937(0.801,1.096) | 0.417 | 0.938(0.738,1.193) | 0.597 | 0.954(0.746,1.22) | 0.701 |
| **Q3** | 0.923(0.771,1.103) | 0.380 | 0.899(0.762,1.06) | 0.205 | 0.912(0.719,1.158) | 0.445 | 0.871(0.702,1.081) | 0.206 |
| **Q4** | 0.756(0.611,0.933) | 0.009 | 0.856(0.717,1.02) | 0.082 | 0.761(0.585,0.99) | 0.042 | 0.859(0.684,1.078) | 0.186 |
| **DII continous** | 1.035(0.99,1.082) | 0.131 | 1.047(1.003,1.094) | 0.036 | 1.066(1.002,1.133) | 0.044 | 1.026(0.961,1.094) | 0.44 |
| **DII Category(ref Q1)** |  |  |  |  |  |  |  |  |
| **Q2** | 1.081(0.904,1.293) | 0.392 | 0.936(0.765,1.147) | 0.523 | 1.291(0.994,1.677) | 0.056 | 0.941(0.72,1.229) | 0.649 |
| **Q3** | 1.044(0.865,1.26) | 0.651 | 0.972(0.803,1.181) | 0.777 | 1.138(0.881,1.471) | 0.317 | 0.905(0.709,1.156) | 0.421 |
| **Q4** | 1.149(0.944,1.399) | 0.165 | 1.109(0.92,1.341) | 0.280 | 1.314(0.999,1.728) | 0.051 | 1.007(0.781,1.297) | 0.959 |
| **MEDI continous** | 0.912(0.849,0.979) | 0.011 | 0.913(0.857,0.973) | 0.005 | 0.914(0.826,1.011) | 0.079 | 0.95(0.875,1.032) | 0.225 |
| **MEDI Category(ref Q1)** |  |  |  |  |  |  |  |  |
| **Q2** | 1.008(0.844,1.201) | 0.926 | 0.939(0.798,1.102) | 0.443 | 1.042(0.804,1.349) | 0.755 | 1.036(0.827,1.297) | 0.755 |
| **Q3** | 0.863(0.73,1.018) | 0.082 | 0.917(0.792,1.061) | 0.246 | 0.882(0.703,1.105) | 0.27 | 0.918(0.77,1.095) | 0.337 |
| **Q4** | 0.729(0.561,0.937) | 0.016 | 0.766(0.623,0.938) | 0.011 | 0.633(0.437,0.918) | 0.017 | 0.887(0.661,1.19) | 0.418 |
| **DASHI continous** | 0.935(0.879,0.994) | 0.031 | 0.956(0.908,1.007) | 0.093 | 0.908(0.835,0.987) | 0.024 | 0.96(0.889,1.036) | 0.287 |
| **DASHI Category(ref Q1)** |  |  |  |  |  |  |  |  |
| **Q2** | 0.943(0.798,1.114) | 0.49 | 0.923(0.787,1.083) | 0.328 | 0.837(0.682,1.027) | 0.087 | 0.893(0.719,1.108) | 0.298 |
| **Q3** | 0.776(0.646,0.931) | 0.007 | 0.954(0.811,1.123) | 0.573 | 0.739(0.587,0.932) | 0.011 | 0.934(0.762,1.146) | 0.509 |
| **Q4** | 0.834(0.681,1.018) | 0.075 | 0.815(0.687,0.967) | 0.019 | 0.765(0.581,1.007) | 0.056 | 0.843(0.656,1.084) | 0.180 |

**Model 1**: age group, race, income, education, marital

**Model 2**:age group, race, income, education, marital, BMI, smoking, drinking, physical activity.

**Model 3**: age group, race, income, education, marital, BMI, smoking, drinking, physical activity, diabetes, hypertension.

**HEI**: Healthy Eating Index; **DII**: Dietary Inflammation Index; **MEDI**: Mediterranean Diet Index; **DASHI**: Dietary Approaches to Stop Hypertension Index; ***P***: p value for model 3

**Supplement Table 3** Relationship between different dietary indices and emphysema among subgroup by gender.

| **Variable** | **OR (95%CI) simple without weighted** | | | | **OR (95%CI) simple with weighted** | | | |
| --- | --- | --- | --- | --- | --- | --- | --- | --- |
|  | **Male** | ***P*** | **Female** | ***P*** | **Male** | ***P*** | **Female** | ***P*** |
| **HEI-2020 continous** | 0.982(0.971,0.993) | 0.001 | 0.983(0.969,0.996) | 0.009 | 0.98(0.965,0.995) | 0.008 | 0.996(0.979,1.014) | 0.677 |
| **HEI-2020 Category(ref Q1)** |  |  |  |  |  |  |  |  |
| **Q2** | 0.086(0.165,0.52) | 0.603 | -0.045(0.205,-0.218) | 0.827 | 1.017(0.633,1.634) | 0.945 | 1.249(0.789,1.978) | 0.338 |
| **Q3** | -0.241(0.186,-1.296) | 0.195 | -0.136(0.218,-0.623) | 0.533 | 0.924(0.551,1.551) | 0.763 | 0.987(0.592,1.645) | 0.959 |
| **Q4** | -0.821(0.25,-3.291) | 0.001 | -0.72(0.28,-2.574) | 0.01 | 0.354(0.173,0.727) | 0.005 | 0.817(0.415,1.608) | 0.553 |
| **DII continous** | 1.158(1.052,1.277) | 0.003 | 1.185(1.044,1.351) | 0.01 | 1.202(1.044,1.384) | 0.011 | 1.168(1.002,1.361) | 0.047 |
| **DII Category(ref Q1)** |  |  |  |  |  |  |  |  |
| **Q2** | 1.151(0.755,1.765) | 0.516 | 1.61(0.757,3.725) | 0.236 | 1.463(0.802,2.669) | 0.212 | 1.648(0.656,4.139) | 0.283 |
| **Q3** | 1.596(1.067,2.415) | 0.025 | 2.392(1.216,5.273) | 0.018 | 1.959(1.031,3.721) | 0.04 | 2.026(0.893,4.595) | 0.09 |
| **Q4** | 1.52(1.01,2.316) | 0.047 | 2.355(1.214,5.145) | 0.019 | 1.977(1.05,3.722) | 0.035 | 1.964(0.871,4.432) | 0.103 |
| **MEDI continous** | 0.773(0.67,0.891) | <0.001 | 0.714(0.597,0.851) | <0.001 | 0.77(0.625,0.95) | 0.016 | 0.841(0.698,1.014) | 0.069 |
| **MEDI Category(ref Q1)** |  |  |  |  |  |  |  |  |
| **Q2** | 0.894(0.629,1.254) | 0.524 | 0.755(0.493,1.134) | 0.185 | 1.029(0.623,1.7) | 0.911 | 0.878(0.469,1.644) | 0.68 |
| **Q3** | 0.526(0.363,0.749) | 0 | 0.618(0.405,0.926) | 0.022 | 0.672(0.384,1.177) | 0.162 | 0.949(0.554,1.628) | 0.848 |
| **Q4** | 0.516(0.29,0.866) | 0.017 | 0.354(0.168,0.671) | 0.003 | 0.381(0.178,0.813) | 0.013 | 0.457(0.227,0.921) | 0.029 |
| **DASHI continous** | 0.857(0.757,0.967) | 0.013 | 0.918(0.794,1.058) | 0.243 | 0.871(0.737,1.03) | 0.104 | 0.996(0.849,1.167) | 0.955 |
| **DASHI Category(ref Q1)** |  |  |  |  |  |  |  |  |
| **Q2** | 1.191(0.862,1.646) | 0.289 | 1.313(0.862,2.015) | 0.208 | 1.219(0.759,1.959) | 0.407 | 1.702(1.08,2.681) | 0.023 |
| **Q3** | 0.859(0.593,1.235) | 0.417 | 1.208(0.775,1.89) | 0.405 | 1.027(0.571,1.849) | 0.928 | 1.539(0.893,2.652) | 0.119 |
| **Q4** | 0.588(0.368,0.914) | 0.022 | 0.789(0.473,1.3) | 0.356 | 0.608(0.316,1.17) | 0.134 | 1.275(0.696,2.337) | 0.426 |

**Model 1**: age group, race, income, education, marital

**Model 2**:age group, race, income, education, marital, BMI, smoking, drinking, physical activity.

**Model 3**: age group, race, income, education, marital, BMI, smoking, drinking, physical activity, diabetes, hypertension.

**HEI**: Healthy Eating Index; **DII**: Dietary Inflammation Index; **MEDI**: Mediterranean Diet Index; **DASHI**: Dietary Approaches to Stop Hypertension Index; ***P***: p value for model 3

**Supplement Table 4** Relationship between different dietary indices and CB among subgroup by gender.

| **Variable** | **OR (95%CI) simple without weighted** | | | | **OR (95%CI) simple with weighted** | | | |
| --- | --- | --- | --- | --- | --- | --- | --- | --- |
|  | **Male** | ***P*** | **Female** | ***P*** | **Male** | ***P*** | **Female** | ***P*** |
| **HEI-2020 continous** | 0.997(0.988,1.005) | 0.429 | 0.985(0.978,0.992) | <0.001 | 1.002(0.99,1.014) | 0.741 | 0.983(0.974,0.992) | <0.001 |
| **HEI-2020 Category(ref Q1)** |  |  |  |  |  |  |  |  |
| **Q2** | 0.941(0.72,1.228) | 0.656 | 0.797(0.645,0.984) | 0.035 | 0.999(0.725,1.377) | 0.997 | 0.754(0.566,1.005) | 0.054 |
| **Q3** | 0.974(0.732,1.29) | 0.856 | 0.781(0.624,0.975) | 0.03 | 1.228(0.825,1.829) | 0.307 | 0.732(0.537,0.999) | 0.049 |
| **Q4** | 0.91(0.651,1.259) | 0.574 | 0.575(0.444,0.741) | 0 | 1.054(0.642,1.73) | 0.832 | 0.577(0.396,0.841) | 0.005 |
| **DII continous** | 1.021(0.951,1.098) | 0.562 | 1.104(1.037,1.175) | 0.002 | 1.012(0.922,1.111) | 0.797 | 1.127(1.03,1.232) | 0.01 |
| **DII Category(ref Q1)** |  |  |  |  |  |  |  |  |
| **Q2** | 1.096(0.814,1.478) | 0.547 | 1.154(0.847,1.582) | 0.369 | 1.297(0.868,1.937) | 0.2 | 1.271(0.798,2.024) | 0.308 |
| **Q3** | 1.139(0.843,1.544) | 0.398 | 1.277(0.956,1.723) | 0.103 | 1.158(0.743,1.806) | 0.511 | 1.089(0.724,1.638) | 0.678 |
| **Q4** | 1.015(0.74,1.393) | 0.927 | 1.455(1.1,1.947) | 0.01 | 0.977(0.641,1.491) | 0.915 | 1.579(1.03,2.423) | 0.037 |
| **MEDI continous** | 1.013(0.905,1.133) | 0.822 | 0.833(0.761,0.91) | 0 | 1.121(0.97,1.297) | 0.121 | 0.85(0.75,0.963) | 0.011 |
| **MEDI Category(ref Q1)** |  |  |  |  |  |  |  |  |
| **Q2** | 0.974(0.732,1.283) | 0.851 | 0.755(0.601,0.944) | 0.015 | 1.225(0.877,1.709) | 0.23 | 0.707(0.537,0.932) | 0.014 |
| **Q3** | 0.915(0.701,1.188) | 0.51 | 0.784(0.639,0.958) | 0.018 | 1.118(0.764,1.636) | 0.561 | 0.737(0.555,0.98) | 0.036 |
| **Q4** | 0.883(0.58,1.308) | 0.549 | 0.56(0.41,0.753) | 0 | 1.125(0.63,2.01) | 0.687 | 0.652(0.411,1.034) | 0.069 |
| **DASHI continous** | 0.938(0.852,1.032) | 0.191 | 0.906(0.842,0.975) | 0.009 | 0.904(0.788,1.037) | 0.148 | 0.938(0.845,1.04) | 0.22 |
| **DASHI Category(ref Q1)** |  |  |  |  |  |  |  |  |
| **Q2** | 1.043(0.753,1.445) | 0.801 | 0.953(0.73,1.243) | 0.723 | 0.85(0.574,1.259) | 0.413 | 0.99(0.749,1.31) | 0.945 |
| **Q3** | 0.808(0.557,1.164) | 0.256 | 0.818(0.613,1.088) | 0.17 | 0.729(0.48,1.107) | 0.136 | 0.865(0.643,1.166) | 0.337 |
| **Q4** | 0.783(0.508,1.186) | 0.257 | 0.585(0.415,0.816) | 0.002 | 0.728(0.486,1.088) | 0.12 | 0.851(0.611,1.185) | 0.334 |

**Model 1**: age group, race, income, education, marital

**Model 2**:age group, race, income, education, marital, BMI, smoking, drinking, physical activity.

**Model 3**: age group, race, income, education, marital, BMI, smoking, drinking, physical activity, diabetes, hypertension.

**HEI**: Healthy Eating Index; **DII**: Dietary Inflammation Index; **MEDI**: Mediterranean Diet Index; **DASHI**: Dietary Approaches to Stop Hypertension Index; ***P***: p value for model 3

**
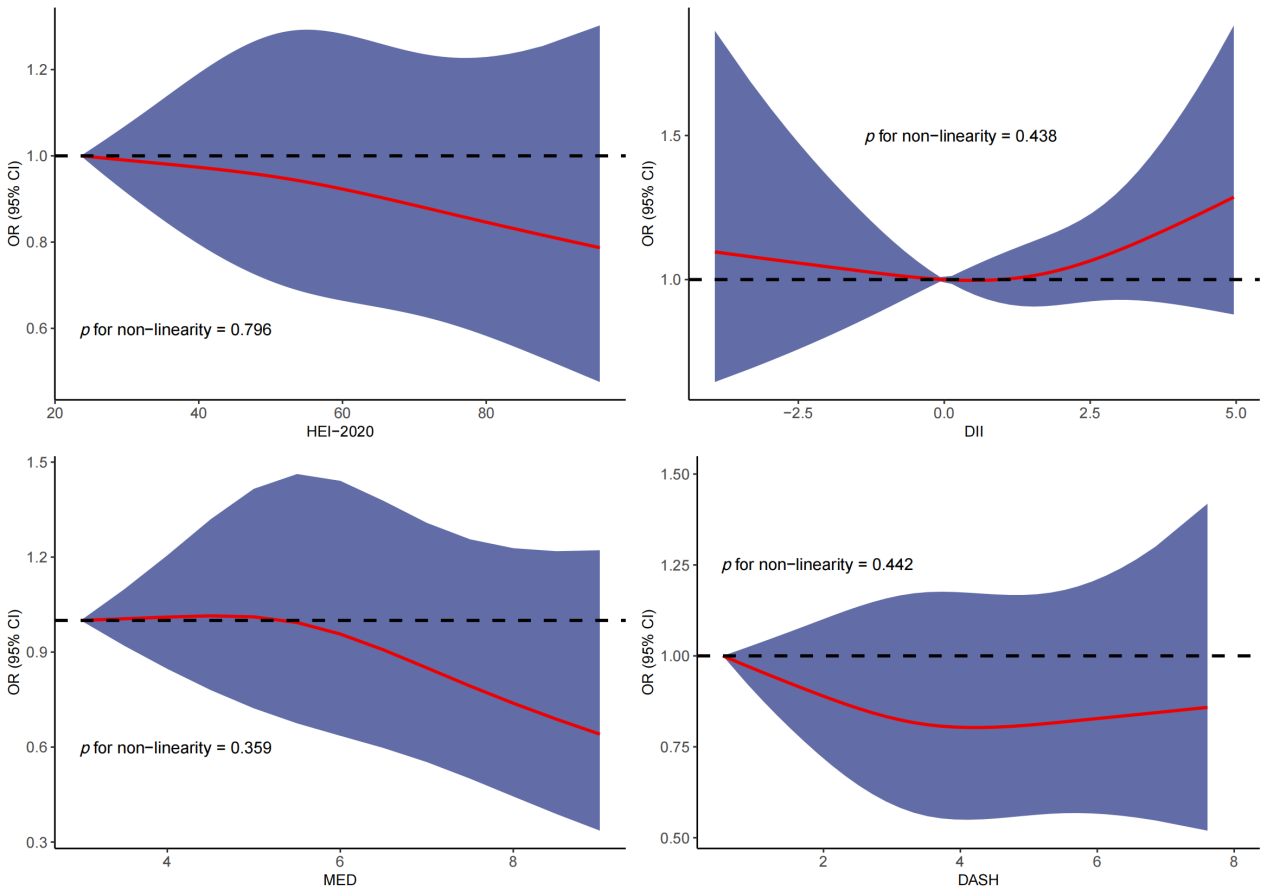
Supplementary Figure 1** Dose–response association between dfour dietary indeices (HEI-2020, DII ,MED and DASH) in continues and asthma using restricted cubic splines(RCS) in the US adult male population. All models adjusted for age group, race, income, education, marital, BMI, smoking, drinking, physical activity, diabetes, hypertension.

**
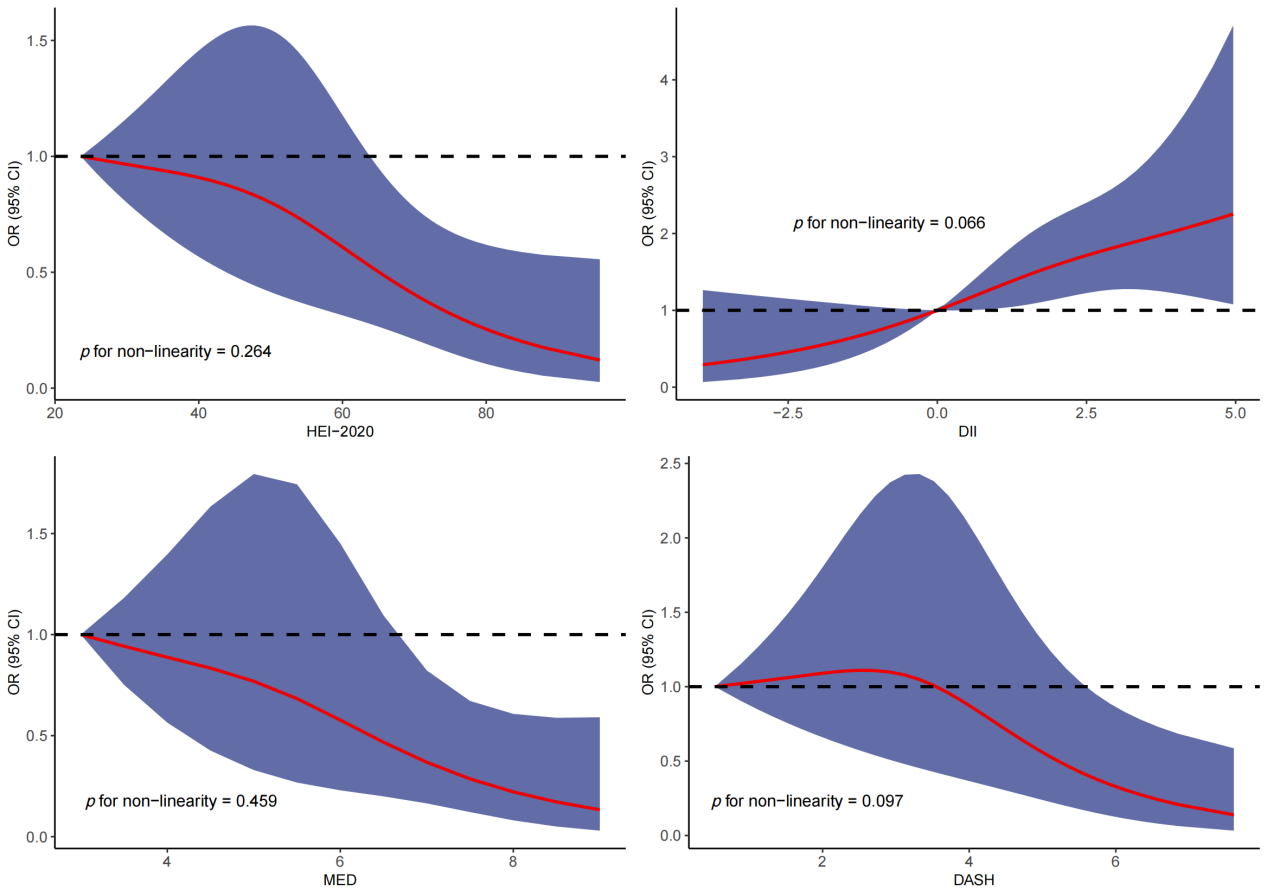
Supplementary Figure 2** Dose–response association between four dietary indeices (HEI-2020, DII ,MED and DASH) in continues and emphysema using restricted cubic splines(RCS) in the US adult male population. All models adjusted for age group, race, income, education, marital, BMI, smoking, drinking, physical activity, diabetes, hypertension.

**
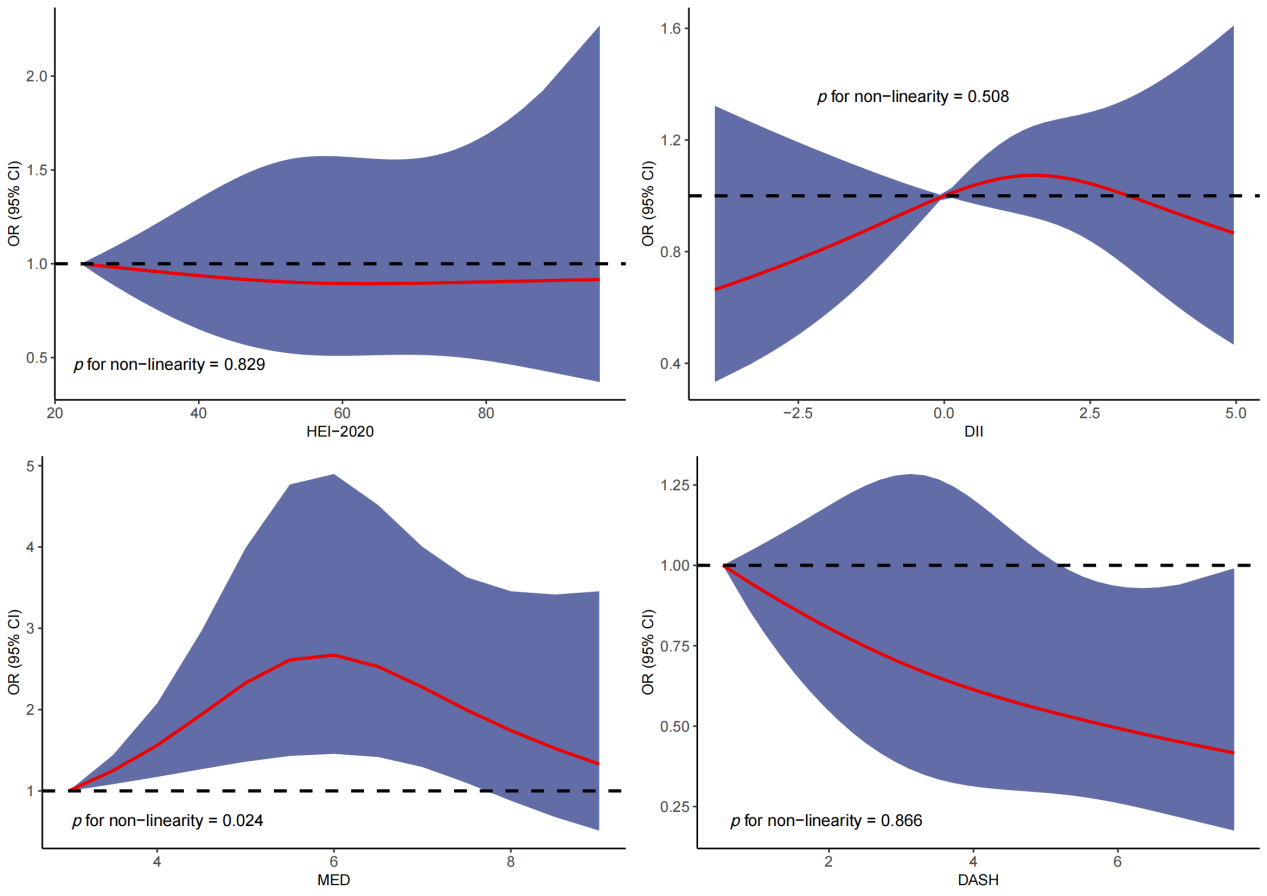
Supplementary Figure 3** Dose–response association between four dietary indeices (HEI-2020, DII ,MED and DASH) in continues and chronic bronchitis(CB) using restricted cubic splines(RCS) in the US adult male population. All models adjusted for age group, race, income, education, marital, BMI, smoking, drinking, physical activity, diabetes, hypertension.

**
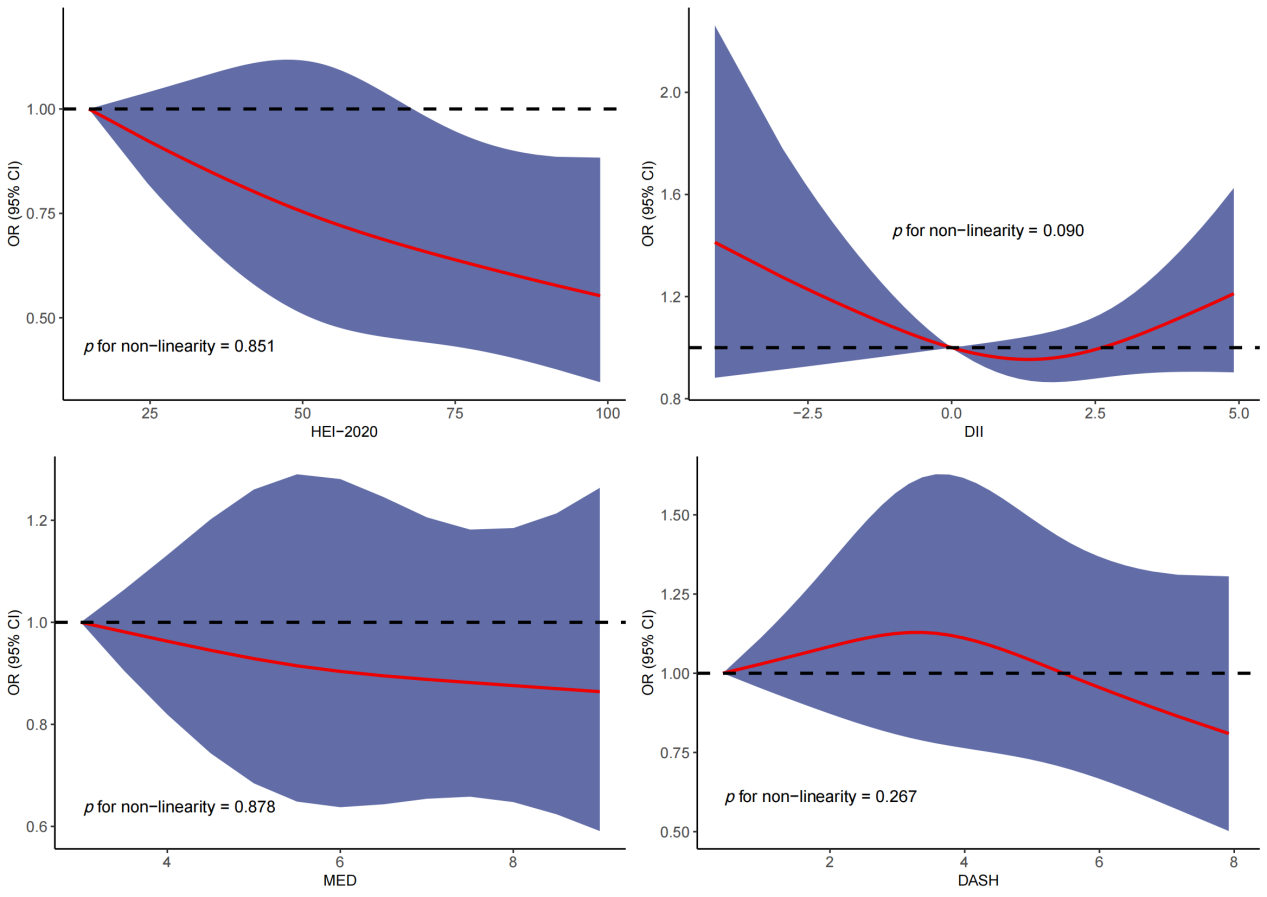
Supplementary Figure 4** Dose–response association between four dietary indeices (HEI-2020, DII ,MED and DASH) in continues and asthma using restricted cubic splines(RCS) in the US adult female population. All models adjusted for age group, race, income, education, marital, BMI, smoking, drinking, physical activity, diabetes, hypertension.

**
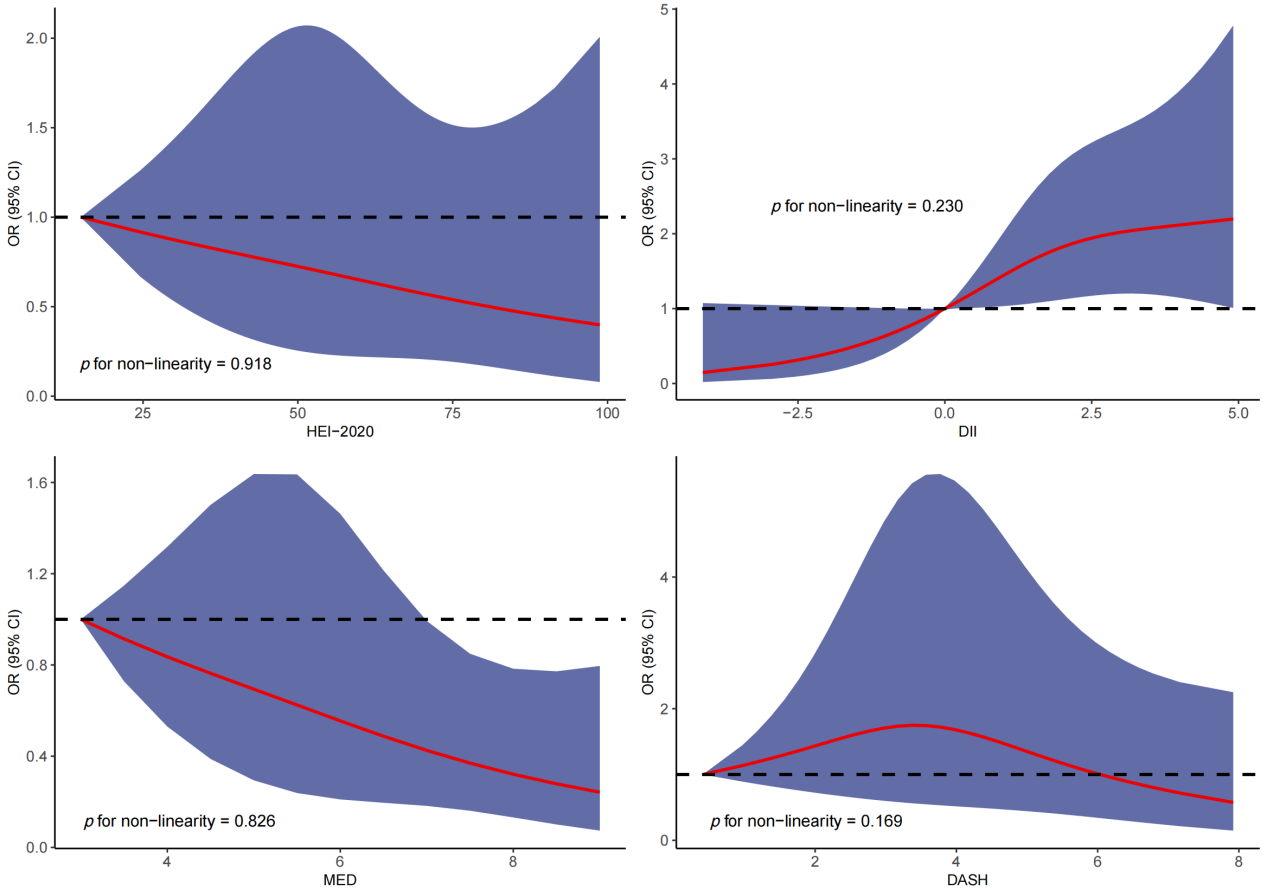
Supplementary Figure 5** Dose–response association between different four dietary indeices (HEI-2020, DII ,MED and DASH) in continues and emphysema using restricted cubic splines(RCS) in the US adult female population. All models adjusted for age group, race, income, education, marital, BMI, smoking, drinking, physical activity, diabetes, hypertension.

**
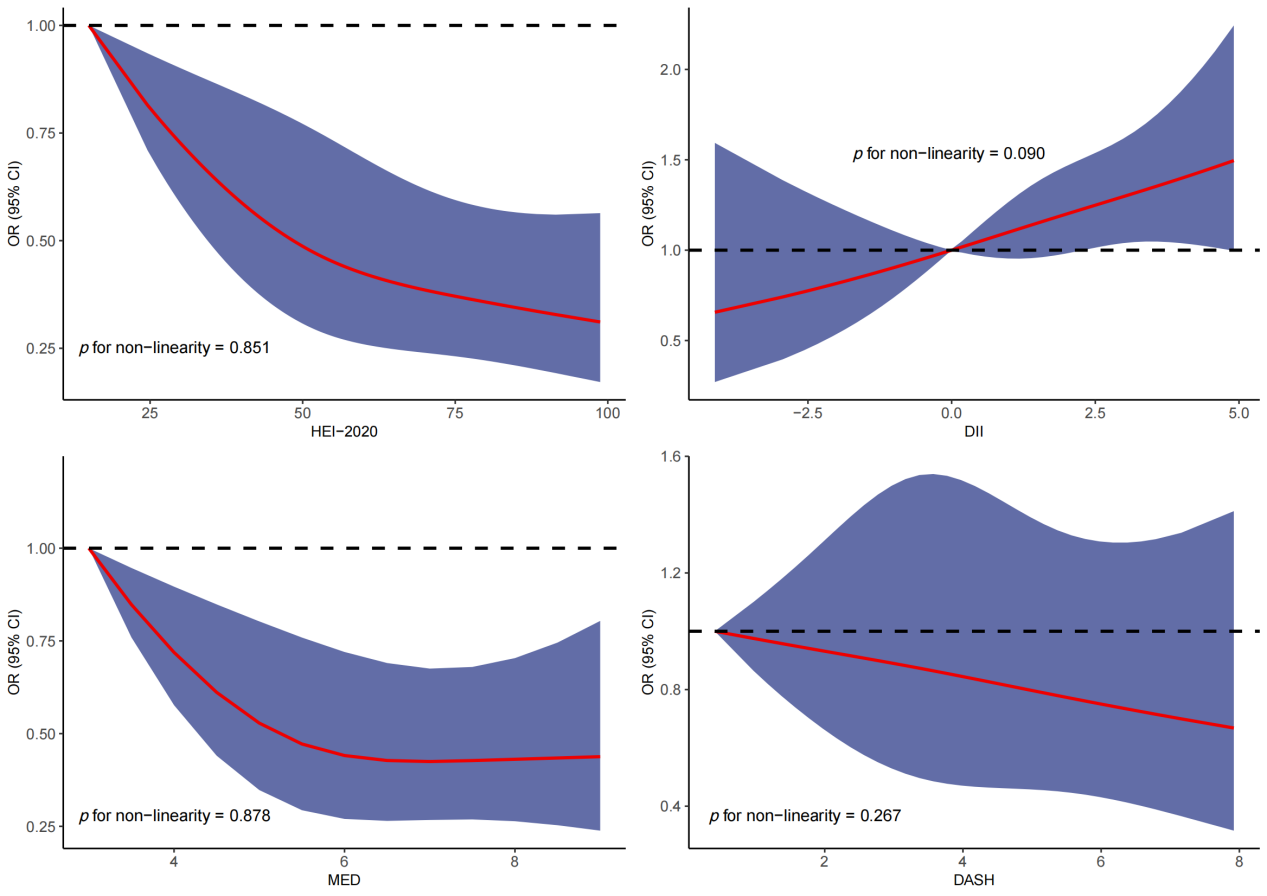
Supplementary Figure 6** Dose–response association betweenfour dietary indeices (HEI-2020, DII ,MED and DASH) in continues and chronic bronchitis(CB) using restricted cubic splines(RCS) in the US adult female population. All models adjusted for age group, race, income, education, marital, BMI, smoking, drinking, physical activity, diabetes, hypertension.

# Reference

1. Shams-White, M. M. *et al.* Healthy Eating Index-2020: Review and Update Process to Reflect the Dietary Guidelines for Americans,2020-2025. *J Acad Nutr Diet* **123**, 1280–1288 (2023).

2. Shivappa, N., Steck, S. E., Hurley, T. G., Hussey, J. R. & Hébert, J. R. Designing and developing a literature-derived, population-based dietary inflammatory index. *Public Health Nutr* **17**, 1689–1696 (2014).

3. Trichopoulou, A., Costacou, T., Bamia, C. & Trichopoulos, D. Adherence to a Mediterranean diet and survival in a Greek population. *N Engl J Med* **348**, 2599–2608 (2003).

4. Fung, T. T. *et al.* Mediterranean diet and incidence of and mortality from coronary heart disease and stroke in women. *Circulation* **119**, 1093–1100 (2009).

5. Mellen, P. B., Gao, S. K., Vitolins, M. Z. & Goff, D. C., Jr. Deteriorating Dietary Habits Among Adults With Hypertension: DASH Dietary Accordance, NHANES 1988-1994 and 1999-2004. *Archives of Internal Medicine* **168**, 308–314 (2008).
